# Genome-Wide Profiling of P450 Gene Expression Reveals Caste-Specific and Developmental Patterns in *Solenopsis invicta*

**DOI:** 10.3390/ijms26073212

**Published:** 2025-03-30

**Authors:** Ting Li, Feng Liu, Dylan J. Brown, Nannan Liu

**Affiliations:** Department of Entomology and Plant Pathology, Auburn University, Auburn, AL 36849, USA; tli@alasu.edu (T.L.); liufeng@szbl.ac.cn (F.L.); djb0094@auburn.edu (D.J.B.)

**Keywords:** *Solenopsis invicta*, cytochrome P450s, development, stages and castes differentiation, reproduction, Halloween genes

## Abstract

P450 enzymes are integral to insect physiology, metabolism, hormone regulation, and adaptation to environmental challenges. By leveraging transcriptomic and genomic data, this study characterized the expression of 68 unique P450 genes across developmental stages and castes in the red imported fire ant (*Solenopsis invicta*), uncovering stage- and caste-specific differential expression patterns. Genes from the CYP4, CYP6, and CYP9 families, known for metabolizing exogenous and endogenous compounds, were highly expressed in early larval stages and minim workers, underscoring their roles in supporting rapid growth, hormone metabolism, colony maintenance, and brood care. The overexpression of *CYP4AA1*—linked to pheromone production—in queens, female alates, and female alate pupae highlights its critical functions in reproductive dominance, social structure maintenance, and colony dynamics. Here, juvenile hormone biosynthesis genes, including *CYP305A1* and *CYP315A1*, exhibited significant overexpression in later instar larvae and larger workers, emphasizing their roles in development and in fulfilling colony-wide physiological demands. The “Halloween genes” (*CYP302A1*, *CYP306A1*, *CYP315A1*, *CYP307A1*, and *CYP314A1*) and *CYP18A1* demonstrated dynamic regulation across developmental stages and castes, reflecting their essential contributions to hormonal production and balance throughout *S. invicta*’s lifecycle. These findings offer valuable insights into the molecular and biological mechanisms driving *S. invicta*’s social organization, developmental transitions, physiological adaptations, and evolutionary success. They also provide a foundation for future research into the regulatory pathways governing P450 gene expression and function.

## 1. Introduction

Cytochrome P450 enzymes (P450s) are a diverse superfamily of heme-thiolate proteins found in the genomes of virtually all organisms [1]. P450s play critical roles in the metabolism of endogenous compounds, including hormones, fatty acids, and steroids, which are essential for insect development, reproduction, growth, and behavior [2]. These functions underscore the importance of P450s in physiological processes where hormones and endogenous chemical signaling are fundamental. Additionally, P450s are involved in the metabolism of exogenous compounds such as drugs, pesticides, and environmental chemicals, highlighting their significance in detoxification and adaptation [3,4,5]. Variations in P450 expression, whether baseline or upregulated, can substantially influence the pharmacological, toxicological, and physiological outcomes of both xenobiotics and endogenous compounds [6,7,8,9]. Advances in transcriptome and genome sequencing have identified a vast number of P450 genes across animals, plants, fungi, and bacteria [10], significantly enhancing our understanding of their roles and functions. In insects, P450s are integral to the biosynthesis and degradation of endogenous compounds, including pheromones, 20-hydroxyecdysone, and juvenile hormone (JH), which regulate growth, development, reproduction, and sex determination [11,12,13,14].

A distinguishing feature of insect P450s is their tissue-specific and caste-specific differential expression, which modulates enzyme levels and activity for hormone metabolism, detoxification pathways, and developmental processes. Studies have highlighted the roles of P450 enzymes in the caste differentiation of social insects, where they regulate hormone levels, chemical communication, and pheromone synthesis and release [15,16,17,18,19]. These findings demonstrate the diverse functions of P450 enzymes in insect physiology, particularly in development, reproduction, and the complex social organization of eusocial species. Understanding the regulatory mechanisms of P450 enzymes is essential for unraveling the molecular basis of caste differentiation, social behavior, and hormonal regulation in social insects, providing insights for the sustainable management of eusocial insect populations in both beneficial and pest contexts.

As with other social insects, the red imported fire ant (*S. invicta*) is a highly invasive species exhibiting remarkable polyphenism. Its distinct castes—queens, workers, and alates—perform specialized roles that are critical for colony success, adaptation, and maintenance. In many insect species, differential gene expression, rather than genetic polymorphism, drives polyphenism, influencing caste differentiation, reproduction, and social hierarchy [20,21,22,23]. Despite its significance as a model social insect, the role of P450s in *S. invicta* physiology remains poorly understood. While previous studies [24] have identified worker-specific expression of *CYP4AB1* and *CYP4AS1* in *S. invicta*, suggesting their involvement in unique physiological functions, the full spectrum of P450-mediated processes, including development, caste differentiation, reproduction, and colony maintenance, has not been comprehensively explored. Compared to other insect species, research on the roles of P450 genes in *S. invicta*, particularly in queen-specific biology and critical physiological processes, is notably limited. This gap highlights the urgent need for focused investigations to elucidate the molecular mechanisms driving the species’ social and physiological adaptations.

This study aims to address these gaps by leveraging transcriptomic and genomic data to explore the roles of P450 enzymes in *S. invicta*’s social structure, development, caste differentiation, and colony maintenance. For the first time, we have comprehensively characterized the expression levels of 68 unique P450 genes identified in the *S. invicta* genome across all life stages and castes. Expression profiles were compared across developmental stages and castes, including male alates versus female alates, females versus queens, 1st–2nd instar larvae versus 3rd–4th instar larvae, small workers versus large workers, large larvae versus alate pupae, alate pupae versus alates, and large larvae versus worker pupae. By elucidating the expression patterns of P450 genes across castes and developmental stages, this research seeks to uncover the molecular underpinnings of *S. invicta*’s complex social organization and physiological processes.

## 2. Results

### 2.1. Distribution of Cytochrome P450 Genes Across Families and Clans in S. invicta

**Figure 1:**
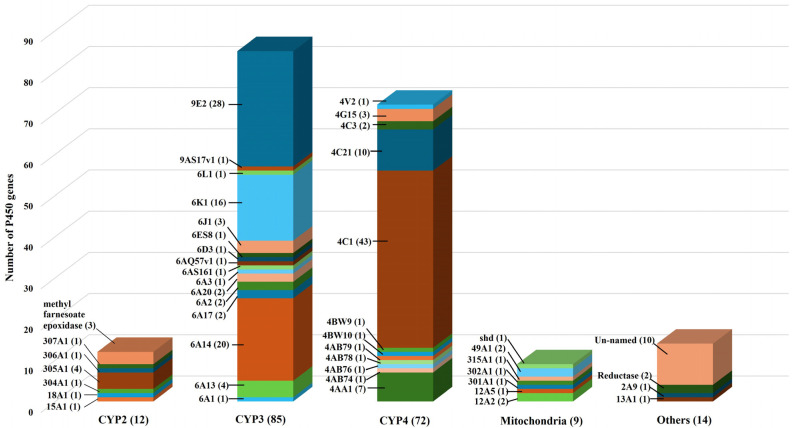
Distribution of Cytochrome P450 Genes Across Families and Clans in the Red Imported Fire Ant *S. invicta*. The P450s are organized into four major clans, along with 14 additional genes not assigned to specific clans. The columns represent the number of P450 genes in each family, grouped according to their respective clans. The P450 gene sequence information generated is from the genome database of the *S. invicta* genome sequence (https://www.ncbi.nlm.nih.gov/datasets/genome/GCF_016802725.1/; https://hymenoptera.elsiklab.missouri.edu/, accessed on 1 December 2024).

Genome sequencing analyses identified 192 unique P450s in the genome of the fire ant, *S. invicta* [25,26]. Of these, 182 genes were functionally annotated, while 10 remained unannotated. Based on the characterization of insect P450s [3], the full-length *S. invicta* P450 genes were categorized into four major clans: CYP2, CYP3, CYP4, and mitochondrial. Additionally, other specific P450 genes were identified, including two reductases, one *CYP2A9*, and *CYP13A1* (Figure 1). Among the 182 functionally annotated *S. invicta* P450 genes, the majority belong to the CYP3 and CYP4 clans.

The CYP3 clan encompasses 85 P450 genes distributed across two CYP families: the CYP6 family (56 genes) and the CYP9 family (29 genes). The CYP4 clan, in contrast, is represented by a single family, the CYP4 family, which comprises 72 P450 genes. The CYP2 clan includes 12 P450 genes spread across several families, including CYP15, CYP18, CYP304, CYP305, CYP306, CYP307, and methyl farnesoate epoxidase. The mitochondrial clan contains nine P450 genes, distributed as follows: three in the CYP12 family, two in CYP49, and one each in CYP301, CYP302, CYP315, and shd (CYP314A1, ecdysone). The diverse array of P450 genes in *S. invicta* reflects the functional versatility commonly observed in insects, underscoring their potential roles in various physiological processes. These include metabolism and detoxification, pheromone production, hormone biosynthesis, reproduction, and environmental adaptation.

### 2.2. P450 Gene Expression Profile in S. invicta

To explore the roles of P450 genes in development, caste differentiation, and reproduction, we selected 68 that comprehensively represent all known clans and families within *S. invicta* to conduct gene expression analysis across the different life stages and castes of fire ants. This selection ensures broad coverage of the P450 superfamily while maintaining experimental feasibility. Expression profiles were examined across nine developmental stages and castes of fire ants, including 1st–2nd instar larvae, 3rd–4th instar larvae, minim workers, big workers, male alates, female alates, queens, worker pupae, and male/female alate pupae. Male alates, which exhibited the lowest expression levels of several P450 genes across all clans in preliminary tests, were used as the baseline for gene expression comparisons to standardize expression levels. P450 gene expression in other stages and castes was quantified relative to the expression levels of the same genes in male alates using RT-qPCR. The NCBI gene names, accession numbers (https://www.ncbi.nlm.nih.gov/gene/, accessed on 1 December 2024), and RT-qPCR primers used for the selected P450 genes are provided in Table S1. Genes that comprehensively represent all known clans and families within *Solenopsis invicta* were represented, ensuring broad coverage of the P450 superfamily while maintaining experimental feasibility.

#### 2.2.1. Comparative Analysis of P450 Gene Expression in Female and Male Alates of Fire Ants

**Figure 2:**
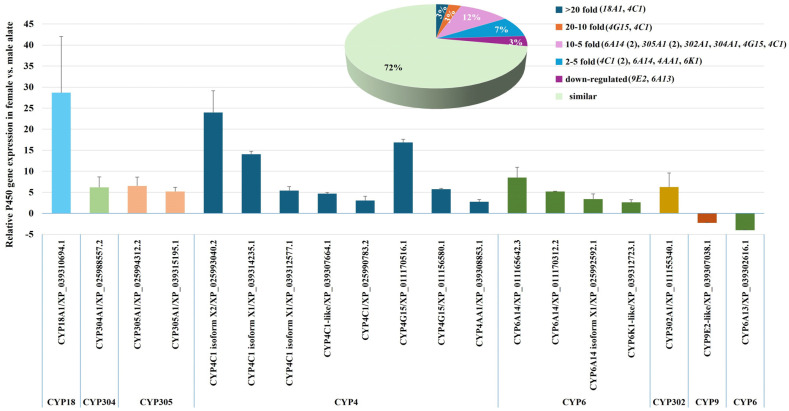
Differential Expression of P450 Genes in Female and Male Alates of *S. invicta*. The expression levels of P450 genes were analyzed and compared between female and male alates. The column chart presents the relative expression ratios of individual P450 genes in females compared to males. The accompanying pie chart visually represents the distribution of upregulated P450 gene expression profiles in females relative to males, with percentages indicating the proportion of upregulated genes within each range.

To investigate the physiological roles of P450 genes in fire ants across different sexual statuses, the expression profiles of 68 selected P450 genes were compared between female and male alates. The analysis identified 17 P450 genes that were upregulated in female alates relative to male alates. These genes were distributed across the following families: eight in the CYP4 family, four in CYP6, two in CYP305, and one each in CYP302, CYP304, and CYP18 (Figure 2, Table S2). The upregulated genes exhibited significant variation in expression levels. Two genes, *CYP18A1* and *CYP4C1* (XP_025993040.2), displayed ≥20-fold-higher transcription levels. Another two genes, *CYP4G15* (XP_011170516.1) and *CYP4C1* (XP_039314235.1), exhibited ≥10- and <20-fold-higher transcription levels. Eight genes, including two variants of *CYP6A14* (XP_011165642.3 and XP_011170312.2), two variants of *CYP305A1* (XP_025994312.2 and XP_039315195.1), *CYP302A1* (*Disembodied*), *CYP304A1*, *CYP4G15* (XP_011156580.1), and *CYP4C1* (XP_039312577.1), were upregulated by ≥5- and <10-fold. The remaining five genes, comprising two variants of *CYP4C1* (XP_039307664.1 and XP_025990783.2), *CYP6A14* (XP_025992592.1), *CYP4AA1*, and *CYP6K1*, displayed ≥2- and <5-fold-higher expression levels. In contrast, two P450 genes, *CYP9E2* and *CYP6A13*, were downregulated in female alates compared to male alates, with transcription levels reduced by 2.27-fold and 4-fold, respectively (Figure 2, Table S2).

The differential expression patterns of P450 genes observed in this analysis highlight their specialized roles in the reproductive and physiological differences between female and male alates. For instance, *CYP4C1*, *CYP6A14*, *CYP6K1*, and *CYP304A1* are primarily associated with metabolism, detoxification, and hormonal regulation [27]. The gene *CYP4G15* is specifically involved in cuticular hydrocarbon synthesis, a process critical for waterproofing and chemical communication [28]. Similarly, *CYP4AA1* likely plays an essential role in maintaining colony cohesion and social structure in fire ants [29,30,31,32]. Among the upregulated genes, *CYP18A1* was the most highly expressed in female alates, with a ~28.7-fold increase in transcription. This gene is implicated in steroid hormone inactivation, metamorphosis, and the ecdysone synthesis pathway, underscoring its critical role in female development and reproduction [33,34,35,36]. *CYP305A1* is pivotal for juvenile hormone synthesis and degradation, further emphasizing its role in regulating developmental and reproductive processes [37,38,39]. Additionally, *CYP302A1* (*Disembodied*) is essential for molting, ovarian maturation, and the regulation of prothoracicotropic hormone synthesis, processes crucial for reproductive success [40]. In contrast, the two downregulated P450 genes, *CYP9E2* and *CYP6A13*, exhibited higher expression levels in male alates. These genes are primarily associated with detoxification processes, suggesting that male alates have specific requirements for xenobiotic metabolism. This finding further underscores the sex-specific physiological roles of P450 genes in fire ants, reflecting their adaptive significance in differing reproductive and environmental contexts.

#### 2.2.2. Comparative Analysis of P450 Gene Expression in Female Alates and Queens

**Figure 3:**
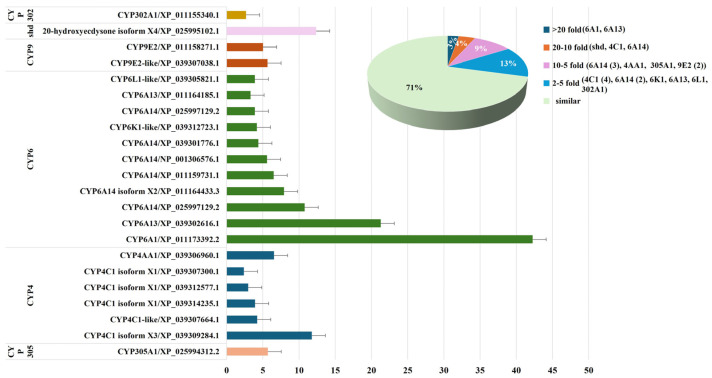
Differential Expression of P450 Genes in Queens Compared to Female Alates of *S. invicta*. The expression levels of P450 genes were analyzed and compared between queens and female alates. The column chart presents the ratios of P450 gene expression levels in queens relative to female alates. Genes are grouped by P450 families, with consistent colors representing genes within the same family. The pie chart illustrates the distribution of upregulated P450 gene expression profiles in queens relative to female alates, with percentages indicating the proportion of upregulated genes within each range.

To investigate the roles of P450 genes in the transition of reproductive status in fire ants, the gene expression profiles of queens and female alates were compared. This analysis revealed 21 P450 genes that were upregulated in queens compared to female alates. These upregulated genes in queens spanned all P450 clans: the CYP4 clan (six genes), including *CYP4AA1* and five variants of *CYP4C1*; the CYP3 clan (12 genes), including 10 genes in CYP6 and two in CYP9; the mitochondrial clan (two genes), including *shd* (*CYP314A1*) and *CYP302A1*; and the CYP2 clan (one gene), *CYP305A1* (Figure 3, Table S3).

Among the 21 P450 genes upregulated in queens, expression levels varied significantly. Two genes, *CYP6A1* and *CYP6A13* (XP_039302616.1), exhibited ≥20-fold-higher transcription levels, both primarily involved in metabolism. Three genes, including *shd* (*CYP314A1*), which is responsible for 20-hydroxyecdysone synthesis [41], as well as *CYP4C1* (XP_039309284.1) and *CYP6A14* (XP_025997129.2), which are associated with metabolism and hormone regulation, exhibited ≥10- and <20-fold-higher transcription levels. Seven genes displayed ≥5- and <10-fold-higher expression levels, including *CYP4AA1*, which is involved in the biosynthesis of the queen’s acid pheromone; three variants of *CYP6A14* (XP_011164433.3, NP_001306576.1, and XP_011159731.1), linked to detoxification and hormone regulation; two variants of *CYP9E2* (XP_011158271.1 and XP_039307038.1), associated with detoxification and metabolism; and *CYP305A1*, which plays a role in juvenile hormone synthesis and degradation. The remaining nine genes exhibited ≥2- and <5-fold-higher transcription levels, including four variants of *CYP4C1* (XP_039307300.1, XP_039314235.1, XP_039312577.1, and XP_039307664.1); *CYP6A14* (XP_039301776.1); *CYP6A13* (XP_011164185.1) and *CYP6K1*, both involved in detoxification and metabolism; *CYP6L1*, which is associated with reproduction [11]; and *CYP302A1* (*Disembodied*), essential for molting, ovarian maturation, and the regulation of prothoracicotropic hormone (Figure 3, Table S3). These findings underscore the significant involvement of P450 genes in the reproductive maturation of fire ants, particularly in the transition from female alates to queens. The results highlight the roles of specific P450 genes in multiple physiological processes, including metabolism, detoxification, hormone regulation, reproduction, and pheromone biosynthesis, all of which are essential for colony maintenance.

#### 2.2.3. Comparative Analysis of P450 Gene Expression in Minim Workers, Big Workers, and Female Alates

Juvenile hormone synthesis and degradation were observed, along with the CYP4 family (three genes), comprising the detoxification genes *CYP4A1* and *CYP4C21*, as well as *CYP4G15*, which mediates cuticle formation, pigmentation, and cuticular hydrocarbon synthesis [28]. The CYP6 family (eight genes) included *CYP6A13* and *CYP6K1*, both associated with metabolism; five variants of *CYP6A14*, which contribute to metabolism and hormone regulation; and *CYP6L1*, linked to reproduction. Other upregulated genes included *CYP9E2*, associated with detoxification and metabolism; methyl farnesoate epoxidase, which contributes to juvenile hormone synthesis and *shd* (*CYP314A1*), also known as ecdysone 20-monooxygenase, which regulates ecdysone synthesis.

**Figure 4:**
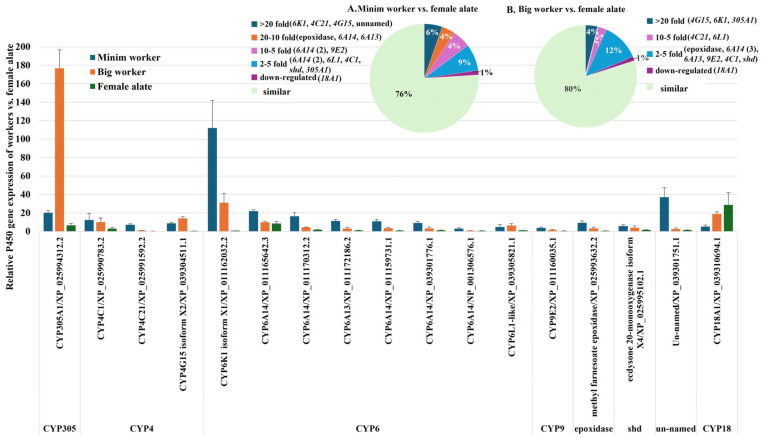
Differential Expression of P450 Genes in Workers Compared to Female Alates of *S. invicta*. RT-qPCR analysis was conducted to assess P450 gene expression levels. The column chart compares P450 gene expression among minim workers, big workers, and female alates, highlighting differentially expressed genes in workers relative to female alates. The pie charts illustrate the distribution of upregulated P450 genes: (**A**) minim workers vs. female alates and (**B**) big workers vs. female alates, with percentages indicating the proportion of upregulated genes within each category.

The expression levels of P450 genes in minim workers compared to female alates varied significantly. Four genes (*CYP6K1*, *CYP4C21*, *CYP4G15*, and the unnamed gene) showed ≥20-fold-higher transcription. Three genes (*methyl farnesoate epoxidase*, *CYP6A14* (XP_011159731.1), and *CYP6A13*) exhibited ≥10- and <20-fold-higher transcription. Three genes demonstrated ≥5- and <10-fold-higher transcription, including two variants of *CYP6A14* (XP_011170312.2 and XP_039301776.1) and *CYP9E2*. Six genes showed ≥2- and <5-fold-higher transcription, including two variants of *CYP6A14* (XP_011165642.3 and NP_001306576.1), *CYP6L1*, *CYP4C1*, *shd* (*CYP314A1*), and *CYP305A1*. Notably, 76% of the P450 genes exhibited similar transcript levels in minim workers and female alates. In big workers, the expression levels of P450 genes compared to female alates were as follows: 4% of the genes (*CYP4G15*, *CYP6K1*, and *CYP305A1*) showed ≥20-fold-higher transcription; 3% (*CYP4C21* and *CYP6L1*) exhibited ≥5- and <10-fold-higher transcription; and 12% (*methyl farnesoate epoxidase*, three variants of *CYP6A14* (XP_011159731.1, XP_011170312.2, and XP_039301776.1), *CYP6A13*, *CYP9E2*, *CYP4C1*, and *shd* (*CYP314A1*)) showed ≥2- and <5-fold-higher transcription. Similarly, 80% of P450 genes displayed comparable transcript abundance in big workers and female alates (Figure 4, Table S4).

These findings reveal the distinct expression patterns of P450 genes between workers and female alates, reflecting their unique roles in caste differentiation within the fire ant colony. The upregulated P450 genes in workers emphasize their involvement in metabolism, detoxification, hormone regulation, and reproduction, aligning with their essential roles in colony maintenance and defense.

#### 2.2.4. Upregulation of P450 Genes During Fire Ant Larval Development

**Figure 5:**
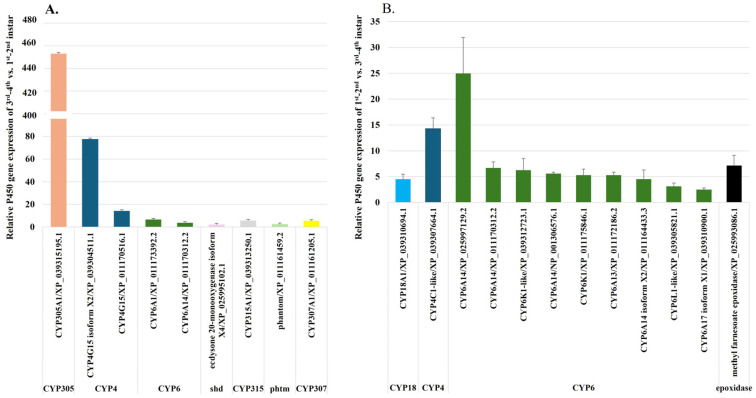
Comparison of P450 Gene Expression Between 1st–2nd and 3rd–4th Instar Larvae of *S. invicta*. P450 gene expression levels were analyzed and compared between 1st–2nd and 3rd–4th instar larvae. (**A**) The upregulated P450 genes in 3rd–4th instar larvae relative to 1st–2nd instar larvae are shown as expression ratios. (**B**) The upregulated P450 genes in 1st–2nd instar larvae relative to 3rd–4th instar larvae are shown as expression ratios.

To investigate the roles of P450 genes in larval development, transcript levels of P450 genes were compared between 1st–2nd instar larvae and 3rd–4th instar larvae of *S. invicta*. The analysis revealed that nine P450 genes were expressed at levels ≥2-fold-higher in 3rd–4th instar larvae compared to 1st–2nd instar larvae. These upregulated genes included *CYP305A1*, which is involved in juvenile hormone synthesis and degradation; two variants of *CYP4G15* (XP_039304511.1 and XP_011170516.1), contributing to cuticular hydrocarbon synthesis; *CYP6A1* and *CYP6A14*, both associated with metabolism; *shd* (*CYP314A1*) and *CYP315A1* (*Shadow*), which are key components of the ecdysone synthesis pathway [41,42]; *phantom* (*CYP306A1*), primarily active in the ecdysteroid biosynthetic pathway [42]; and *spook* (*CYP307A1*), which catalyzes a key step in the ecdysone biosynthesis essential for growth and pupation [13]. Among these, *CYP305A1* and *CYP4G15* (XP_039304511.1) exhibited the highest levels of overexpression in 3rd–4th instar larvae, with fold changes of 453 and 77, respectively (Figure 5A, Table S5A).

Conversely, 12 P450 genes were upregulated in 1st–2nd instar larvae compared to 3rd–4th instar larvae. These included *CYP18A1*, which is involved in pheromone biosynthesis; *CYP4C1*, associated with metabolism; methyl farnesoate epoxidase, which plays a role in juvenile hormone synthesis; and nine *CYP6* genes, including four *CYP6A14* variants (XP_025997129.2, XP_011170312.2, NP_001306576.1, and XP_011164433.3), two *CYP6K1* variants (XP_039312723.1 and XP_011175846.1), *CYP6A13*, *CYP6A17*, and *CYP6L1*. Among these, *CYP6A17* is notable for its role in temperature preference behavior [43], while *CYP6L1* is associated with reproduction (Figure 5B, Table S5B). These findings highlight dynamic changes in P450 gene expression during the larval development of *S. invicta*, underscoring their diverse roles in metabolism, hormone regulation, and physiological adaptation.

#### 2.2.5. P450 Gene Expression During the Developmental Transition from Worker Pupae to Workers

**Figure 6:**
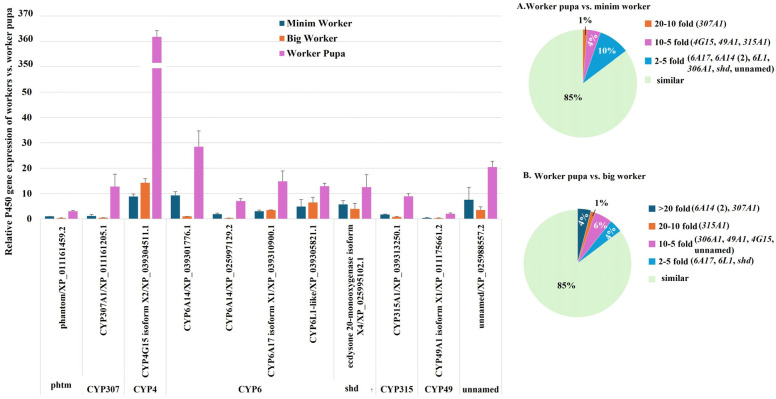
Comparison of P450 Gene Expression Between Worker Pupae, Minim Workers, and Big Workers of *S. invicta*. RT-qPCR analysis was conducted to assess P450 gene expression levels. The column chart presents a comparative analysis of P450 gene expression across worker pupae, minim workers, and big workers, highlighting upregulated genes in worker pupae relative to the other castes. The pie charts depict the distribution of differentially expressed P450 genes: (**A**) worker pupae vs. minim workers and (**B**) worker pupae vs. big workers. Percentages indicate the proportion of upregulated genes within each category.

Our analysis identified significant upregulation of 11 P450 genes in *S. invicta* worker pupae compared to both minim and big workers. These upregulated genes include *phantom* (*CYP306A1*) and *CYP307A1* (*Spook*), which are key players in the ecdysone synthesis pathway; *CYP4G15*, involved in cuticular hydrocarbon synthesis, cuticle formation, and pigmentation; two variants of *CYP6A14* (XP_039301776.1 and XP_025997129.2) and *CYP49A1*, associated with metabolism; *CYP6A17*, essential for temperature preference behavior; *CYP6L1*, linked to reproduction; *shd* (*CYP314A1*) and *CYP315A1* (*Shadow*), critical for ecdysteroid synthesis, including the production of 20-hydroxyecdysone; and one unnamed gene (Figure 6, Table S6).

Among the P450 genes upregulated in worker pupae compared to minim workers, *CYP307A1* (*Spook*) exhibited ≥20-fold-higher transcription. Three genes *CYP4G15*, *CYP49A1*, and *CYP315A1* (*Shadow*) were upregulated by ≥5- and <10-fold, while seven genes *CYP6A17*, two variants of *CYP6A14* (XP_039301776.1 and XP_025997129.2), *CYP6L1*, *CYP306A1* (*Phantom*), *shd* (*CYP314A1*), and the unnamed gene exhibited ≥2- and <5-fold-higher transcription (Figure 6, Table S6). Similarly, the upregulated P450 genes in worker pupae compared to big workers included three genes *CYP307A1* (*Spook*) and two variants of *CYP6A14* (XP_039301776.1 and XP_025997129.2) showing ≥20-fold-higher transcription. One gene, *CYP315A1* (*Shadow*), exhibited ≥10- and <20-fold-higher transcription. Four genes *CYP306A1* (*Phantom*), *CYP49A1*, *CYP4G15*, and the unnamed gene displayed ≥5- and <10-fold-higher transcription, while three genes *CYP6A17*, *CYP6L1*, and *shd* (*CYP314A1*) showed ≥2- and <5-fold-higher transcription. These results highlight significant changes in P450 gene transcription during the transition from workers to worker pupae, emphasizing their critical roles in metamorphosis and the corresponding physiological adaptations.

#### 2.2.6. Upregulation of P450 Genes in the Transition from Late Instar Larvae 3rd–4th to Worker, Male, and Female Pupae

**Figure 7:**
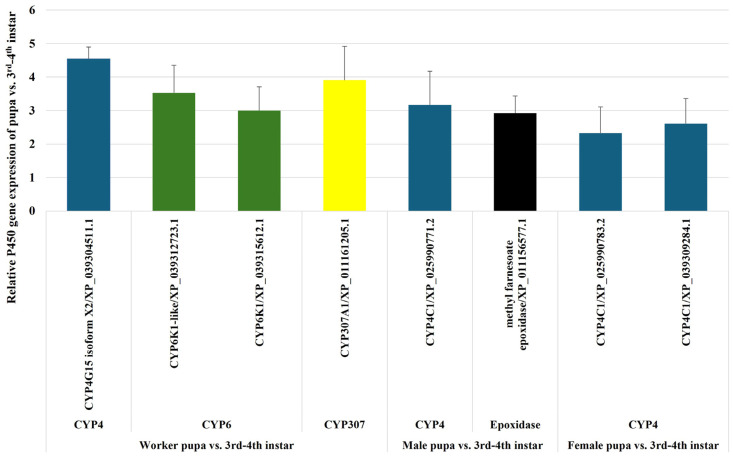
Comparison of P450 Gene Expression in Worker, Male, and Female Pupae Relative to 3rd–4th Instar Larvae of *S. invicta*. P450 gene expression levels were analyzed in three comparisons: worker pupae versus 3rd–4th instar larvae, male pupae versus 3rd–4th instar larvae, and female pupae versus 3rd–4th instar larvae. The column chart presents the relative expression ratios of individual P450 genes in worker, male, and female pupae compared to 3rd–4th instar larvae.

To further investigate the roles of P450 genes in metamorphosis during the pupal stage, we analyzed their transcription levels in 3rd–4th instar larvae, male pupae, female pupae, and worker pupae. The analysis identified eight unique P450 genes that were upregulated in either worker or female/male pupae compared to 3rd–4th instar larvae. In worker pupae, *CYP4G15* was upregulated by 4.5-fold, two variants of *CYP6K1* (XP_039312723.1 and XP_039315612.1) were upregulated by 3.5- and 3-fold, and *CYP307A1* (*Spook*) was upregulated by 3.9-fold. In male pupae, *CYP4C1* exhibited a 3.2-fold increase, and methyl farnesoate epoxidase showed a 2.9-fold increase in transcription levels. In female pupae, two variants of *CYP4C1* (XP_025990783.2 and XP_039309284.1) were upregulated by 2.3- and 2.6-fold compared to the late larval instar, similar to the pattern observed in male pupae (Figure 7, Table S7).

Notably, all three pupal stages exhibited overexpression of *CYP4C1* variants when compared to 3rd–4th instar larvae. This finding suggests that the enhanced expression of *CYP4C1* may play a critical role in managing harmful metabolites, environmental toxins, and endogenous compounds during the pupal stage. Since pupae are often less mobile, this overexpression likely supports detoxification processes and the metabolism of essential endogenous compounds, such as ecdysteroids and juvenile hormones, which are essential for proper developmental transitions. Interestingly, *CYP307A1* (*Spook*), which plays a critical role in the ecdysone synthesis pathway, and *CYP4G15*, involved in cuticular hydrocarbon synthesis contributing to cuticle formation and pigmentation, were upregulated exclusively in worker pupae and not in reproductive male and female alate pupae. Workers undergo significant physiological changes during this stage to develop the robust exoskeleton and musculature required for their labor-intensive tasks, such as foraging and colony defense. This observation highlights the essential role of *CYP4G15* and *CYP307A1* in preparing the worker pupae for cuticle formation, which provides a protective barrier against desiccation and pathogens. Additionally, the cuticle contributes to chemical communication within the colony, a critical function during the pupal stage for workers but less relevant for reproductive forms.

#### 2.2.7. Comparative Analysis of P450 Gene Expression in Fire Ant Pupae and Alates

**Figure 8:**
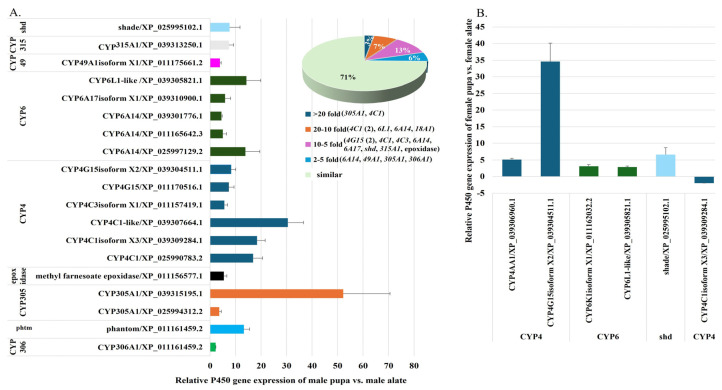
Comparison of P450 gene expression between male pupae and male alates, and between female pupae and female alates of *S. invicta*. (**A**) The column chart shows the relative expression ratios of individual P450 genes in male pupae compared to male alates. The accompanying pie chart visually represents the distribution of upregulated P450 gene expression profiles in male pupae relative to male alates, with percentages indicating the proportion of upregulated genes within each range. (**B**) The column chart shows the relative expression ratios of individual P450 genes in female pupae compared to female alates.

To investigate stage-specific P450 gene expression in *S. invicta*, we analyzed the differential expression of P450 genes in male and female pupae and alates. The analysis revealed that 19 P450 genes were upregulated in male pupae compared to male alates, encompassing a wide range of functions. These included genes involved in metabolism and hormone regulation, such as three *CYP4C1* variants (XP_025990783.2, XP_039309284.1, and XP_039307664.1) and three *CYP6A14* variants (XP_011165642.3, XP_039301776.1, and XP_025997129.2); the ecdysone synthesis pathway, including *Phantom* (*CYP306A1*), *CYP315A1* (*Shadow*), and *shd* (*shade/CYP314A1*); and metamorphosis, represented by *CYP18A1*. Additionally, genes involved in juvenile hormone synthesis and degradation, such as two *CYP305A1* variants (XP_025994312.2 and XP_039315195.1), and genes associated with metabolism and detoxification, including *CYP49A1* and *CYP4C3*, were also upregulated. Other upregulated genes were related to cuticle formation and pigmentation, such as two *CYP4G15* variants (XP_011170516.1 and XP_039304511.1), thermosensation (*CYP6A17*), reproduction (*CYP6L1*), and juvenile hormone synthesis (methyl farnesoate epoxidase) (Figure 8A, Table S8A). Among these genes, *CYP305A1* (XP_039315195.1) and *CYP4C1* (XP_039307664.1) exhibited exceptionally high expression in male pupae, with transcription levels at least 20-fold-higher than in male alates. *CYP305A1* (XP_039315195.1) displayed the highest upregulation, with a 52.21-fold increase. Interestingly, *CYP305A1* (XP_039315195.1) was also upregulated in female alates (Figure 2, Table S2) and in 3rd–4th instar larvae (Figure 5A, Table S5A).

Further analysis revealed moderate upregulation of other genes: Five P450 genes, including two *CYP4C1* variants (XP_025990783.2 and XP_039309284.1), *CYP6A14* (XP_025997129.2), *CYP6L1*, and *CYP18A1*, showed transcription levels 10- to 20-fold higher in male pupae compared to male alates. Eight P450 genes, including two *CYP4G15* variants (XP_011170516.1 and XP_039304511.1), *CYP4C3*, *CYP6A14* (XP_011165642.3), *CYP6A17*, *CYP315A1* (*Shadow*), *shd* (*CYP314A1*), and methyl farnesoate epoxidase, exhibited transcription levels 5- to 10-fold higher. Additionally, four genes, including *CYP6A14* (XP_039301776.1), *CYP49A1*, *CYP305A1*, and *CYP306A1* (*Phantom*), showed 2- to 5-fold-higher transcription. In the comparison between female pupae and female alates, five P450 genes were upregulated in female pupae, while one was upregulated in female alates. For example, *CYP4G15* (XP_039304511.1) showed a 34.59-fold-higher transcription level in male pupae, *CYP6L1* exhibited a 2.88-fold-higher expression, and ecdysone 20-monooxygenase (*shd* (*CYP314A1*)) demonstrated a 6.6-fold-higher expression in male pupae (Figure 8B, Table S8B). In contrast, one variant of *CYP4C1* (XP_039309284.1) was upregulated in female alates. These findings suggest that the upregulated P450 genes play critical roles in developmental processes during the pupal stage in both male and female *S. invicta*. Their involvement in metabolism, hormone regulation, cuticle formation, and reproductive preparation underscores their importance in metamorphosis and the transition to the reproductive caste.

## 3. Discussion

Integrating genomic and transcriptomic data, this study is the first to systematically characterize the functions of P450 genes in *S. invicta*. By analyzing differential expression profiles across life stages and castes, we uncovered the diverse roles of P450 genes in metabolizing toxins, regulating hormones, and processing environmental chemicals. Notably, genes involved in the metabolism of exogenous and endogenous compounds were predominantly from the P450 families CYP4, CYP6, and CYP9—particularly the CYP4C, CYP6A, and CYP9E subfamilies, which were expressed across all castes and stages. Among these, genes such as *CYP4C1*, *CYP6A13*, *CYP6A14*, and *CYP9E1* were particularly abundant, while less common genes like *CYP4C21*, *CYP6K1*, and *CYP6L1* were also detected. Genes in the CYP4 and CYP6 families exhibited high expression in early larval stages (1st–2nd instars) compared to older larvae (3rd–4th instars), reflecting their critical roles in supporting rapid growth, hormone regulation, and detoxification during early development [44,45]. Among adult female castes, the expression of P450 genes varied significantly. Minims (the smallest worker caste) displayed the highest expression levels, followed by large workers, with the lowest levels observed in alates. The elevated P450 activity in minims is likely required to meet their metabolic demands, including maintaining the colony’s internal environment, processing nest-specific chemicals, and engaging in brood care. Their exposure to microbial pathogens, colony waste, and restricted diets necessitates robust detoxification pathways and active P450 enzyme involvement [27].

Large workers exhibited moderate P450 expression levels, consistent with their foraging-related exposure to environmental toxins and relatively mature physiology. In contrast, female alates displayed the lowest expression levels, reflecting their reproductive specialization, limited environmental interaction, and simplified metabolic demands. Their shorter lifespan and primary reproductive focus reduce the need for extensive detoxification pathways [46,47]. Previous studies on *S. invicta* support these findings, demonstrating differential P450 gene expression among worker subcastes—big, medium, and minor workers. Minim workers (minims) exhibited unique P450 expression patterns associated with their specialized roles in brood care and nest maintenance [48]. These variations highlight the adaptability of P450 genes to the specific physiological and environmental demands of each caste and life stage.

Beyond their metabolic functions, P450s play critical roles in communication, development, reproduction, and caste differentiation in social insects. While these roles are well-characterized in *Drosophila*, they remain underexplored in other insect species. P450 enzymes mediate the biosynthesis and degradation of endogenous compounds such as pheromones, ecdysteroids, and juvenile hormone (JH), emphasizing their importance in modulating hormone levels, chemical communication, and pheromone synthesis [16,17,18,19,49].

The interaction between P450-mediated pheromone production and hormone regulation is pivotal for shaping colony dynamics and maintaining social organization. For example, in the termite *Cryptotermes secundus*, the P450 gene *Neofem4* (CYP4 family) is associated with queen-specific pheromone production. Silencing *Neofem4* disrupts the queen’s chemical profile, leading workers to behave as though the queen is absent, emphasizing its role in maintaining social hierarchy [17]. Similarly, in honeybees, *CYP4AA1* is implicated in the biosynthesis of the queen’s acid pheromone, essential for colony cohesion and reproductive dominance [29,30,31,32]. Our findings align with these studies, demonstrating that *CYP4AA1* variants are specifically overexpressed in female alates, female alate pupae, and queens, with the highest expression observed in queens (Table 1). This pattern suggests that *CYP4AA1* variants regulate queen-specific functions in ants, such as promoting colony cohesion, maintaining reproductive dominance, and reinforcing social hierarchy [50]. Additionally, *CYP4AA1* overexpression in adult female alates likely reflects its role in pheromone biosynthesis for mate attraction and colony signaling, while elevated expression in female alate pupae may prepare for pheromone production in adulthood [51,52]. Similar findings in honeybees (*Apis mellifera*) show that *CYP4AA1* is differentially expressed in the mandibular glands (MGs) of queens and workers [31]. However, discrepancies, such as those observed by Wu [30], where *CYP4AA1* expression did not vary significantly among mated queens, queenright workers, and queenless workers, likely arise from differences in experimental design, sampling timing, and the temporal or spatial variability of gene expression.

The role of JH in caste differentiation is well-documented in social insects [13]. Hartfelder [53] identified JH as a key regulator of gene expression pathways in honeybees, driving caste-specific traits that distinguish workers from queens. Similarly, studies in the termite *Reticulitermes flavipes* revealed that *CYP15F1* regulates JH levels, enabling JH-dependent soldier caste differentiation [15]. Furthermore, *CYP15A1* and *CYP305A1* have been implicated as terminal enzymes in JH biosynthesis, catalyzing the final steps of methyl farnesoate epoxidation [37,38,39,54]. In our study, *CYP305A1* and *CYP15A1* variants were highly expressed in various castes and developmental stages, with particularly elevated expression in 3rd–4th instar larvae and larger workers, showing fold changes of 254 and 177, respectively, compared to male alates. This pattern underscores their critical role in supporting larval development and meeting colony-wide physiological demands. During later larval stages, *CYP305A1* ensures sufficient JH production to maintain the larval state and prevent premature metamorphosis [55]. In larger workers, *CYP305A1* is associated with reproductive suppression, energy metabolism, and neural development, facilitating their specialized roles in foraging and colony defense [56]. This aligns with studies in termites where higher JH titers were linked to larger worker differentiation and functional specialization [57].

P450s collectively known as “Halloween genes” (*CYP302A1*, *CYP306A1*, *CYP315A1*, *CYP307A1*, and *CYP314A1*) regulate the biosynthesis of 20-hydroxyecdysone (20E), an essential ecdysteroid hormone [41,58]. These genes catalyze key steps in ecdysteroid biosynthesis, supporting molting, development, reproduction, and caste differentiation in insects [15,18,19,40]. Our findings (Table 1) reveal stage- and caste-specific expression of these genes in *S. invicta*. *CYP307A1* catalyzes an early rate-limiting step in ecdysone biosynthesis, with elevated expression observed in 3rd–4th instar larvae and worker pupae, corresponding to increased ecdysteroid demand for growth and tissue remodeling [13,59]. Similarly, *CYP306A1* and *CYP315A1*, which function downstream in the pathway, were upregulated in late larvae and pupae, reflecting their roles in molting and the development of caste-specific traits. Conversely, lower expression of Halloween genes in female alate pupae reflects reduced hormonal demand during reproductive preparation. For example, *CYP302A1* (*Disembodied*) supports ovarian development and egg production in queens, while *CYP314A1* (*Shade*), which catalyzes ecdysone to 20E, demonstrates broader functionality, supporting both developmental transitions in larvae and ovarian activity in queens.

*CYP18A1*, though not a Halloween gene, regulates ecdysteroid inactivation by fine-tuning ecdysone and 20E levels [33,60]. Its overexpression in most castes prevents excessive hormonal activity, maintaining homeostasis during critical transitions [36]. However, reduced *CYP18A1* expression in worker pupae, alate pupae, and queens allows high ecdysteroid levels to support tissue remodeling, reproductive organ development, and ovarian activity, respectively. This highlights *CYP18A1*’s role in balancing hormonal activity to meet caste-specific physiological needs.

## 4. Materials and Methods

### 4.1. Insect Strain

Fire ants were collected from monogyne colonies in the field in Alabama, USA. Samples from each colony were categorized based on developmental stages and castes, including 1st–early 2nd instar larvae, late 3rd–4th instar larvae [61], minim workers, big workers, male alates, female alates, queens, worker pupae, male alate pupae, and female alate pupae. Classification followed the descriptions provided by Biology—Fire Ants in Tennessee (https://fireants.tennessee.edu/about-fire-ants/biology/, accessed on 1 December 2024).

### 4.2. Identification of S. invicta Genes from Genome Assembly

To identify P450 genes in *S. invicta*, we used the UNIL_Sinv_3.0 (GCF_016802725.1) database, which represents the most complete and up-to-date *S. invicta* genome assembly [25]; (NCBI; Hymenoptera Genome Database). A search for “Cytochrome P450” in this database identified 192 putative P450 genes. To ensure the reliability and representativeness of our selection, we excluded genes labeled as “low quality” or “probable”, focusing on those with well-supported annotations. This filtering approach ensured that the selected P450 genes provided broad coverage of the superfamily while maintaining experimental feasibility. Additionally, we aimed to comprehensively represent all known P450 clans and families within *S. invicta*. Based on these criteria, a total of 68 unique P450 genes were selected for gene expression analysis across different life stages and castes of fire ants.

### 4.3. RNA Extraction and cDNA Preparation

Total RNA was extracted from whole bodies of different life stages and castes, including 1st–2nd instar larvae, 3rd–4th instar larvae, small workers, large workers, male alates, female alates, queens, worker pupae, male alate pupae, and female alate pupae of *S. invicta*, using the acidic guanidine thiocyanate-phenol-chloroform method [62]. Following extraction, 5 µg of total RNA from each sample was treated with DNase I using a TURBO DNA-free kit (Ambion, Austin, TX, USA) to remove any contaminating DNA. RNA was further purified by two successive acid phenol:chloroform (Sigma, St. Louis, MI, USA) (1:1) steps, followed by a final chloroform extraction to remove residual phenol. The RNA was precipitated in ethanol and resuspended in sterile distilled water. Each RNA extraction was repeated three times using samples collected from the same colony.

### 4.4. Reverse Transcription Quantitative Real-Time PCR (RT-qPCR)

Total RNA (0.5 µg per sample) was reverse-transcribed using the Transcriptor First Strand cDNA Synthesis Kit (Roche, Indianapolis, IN, USA) in a total volume of 20 μL. First-strand cDNA was synthesized using a random hexamer primer, following the manufacturer’s instructions. RT-qPCR was performed using the dSYBR Green Master Mix kit (Roche, Pleasanton, CA, USA) on an ABI 7500 Real-Time PCR System (Marshall Scientific, Hampton, NH, USA). Each 15 µL reaction contained 7.5 µL SYBR Green master mix, 1 µL of cDNA, and gene-specific primers for P450 genes (Table S1); at a final concentration of 3–5 µM. All reactions, including a no-template negative control, were performed in triplicate. The thermal cycling protocol consisted of an initial denaturation at 95 °C for 10 min, followed by 40 cycles of 95 °C for 15 s and 60 °C for 1 min. RT-qPCR specificity was confirmed by melting curve analysis using 7500 Software v2.0 [63].

The relative expression levels of P450 genes were calculated using the 2^−ΔΔCT^ method [64], with 18S ribosomal RNA (*18S rRNA*) GenBank accession number: AY334566) serving as the endogenous control for normalization, as it has been shown to be stably expressed across different stages and castes of fire ants [24,65]. Preliminary RT-qPCR experiments showed consistent expression of the *18S rRNA* gene across all life stages and castes of *S. invicta*, supporting its use as an internal reference. Each experiment was repeated three to four times using independent RNA extractions. The statistical significance of gene transcript abundance was calculated using Student’s *t*-test for two-sample comparisons and one-way analysis of variance (ANOVA) for multiple-sample comparisons (SAS v9.1 software). A *p*-value ≤ 0.05 was considered statistically significant. Significant upregulation was defined as a ≥2-fold change in transcript abundance [66].

## 5. Conclusions

This study utilizes publicly available genome and transcriptome data to provide the first comprehensive analysis of P450 gene expression in *S. invicta*. Our findings highlight the critical roles of P450 genes from the CYP4, CYP6, and CYP9 families in metabolizing exogenous and endogenous compounds, alongside distinct caste-specific expression patterns that reflect their adaptability to diverse physiological demands. Furthermore, we show that P450 enzymes, such as *CYP4AA1*, *CYP305A1*, *CYP15A1*, the “Halloween genes” (*CYP302A1*, *CYP306A1*, *CYP315A1*, *CYP307A1*, *CYP314A1*), and *CYP18A1*, play pivotal roles in communication, development, reproduction, and caste differentiation by regulating hormonal pathways like juvenile hormone and ecdysteroid production. These insights contribute to understanding the lifecycle and social structure of *S. invicta* and provide a foundation for future research on their evolutionary significance and applications in pest management.

## Figures and Tables

**Table 1:** Cytochrome P450 gene overexpression levels across castes and developmental stages associated with hormone and pheromone biosynthesis or degradation.

		1st-2nd Instar	3rd-4th Instar	Worker Pupa	♂ Alate Pupa	♀ Alate Pupa	Minim Worker	Big Worker	♀ Alate	Queen
Caste/Stage P450 Gene										
*CYP4AA1* XP_039308853.1		-	-	-	-	-	-	-	2.8	-
*CYP4AA1* XP_039306960.1		-	-	-	-	4.5	-	-	-	5.7
*CYP305A1* XP_025994312.2		-	-	-	3.5	-	20	180	6.5	37
*CYP305A1* XP_039315195.1		-	250	-	52	-	-	-	5.2	-
*Methyl farnesoate epoxidase (CYP15A1*) XP_011156577.1		-	-	-	5.3	-	-	-	-	-
*Methyl farnesoate epoxidase* (*CYP15A1*) XP_025993632.2		-	-	-	-	-	9.3	3.2	-	-
*Methyl farnesoate epoxidase* (*CYP15A1*) XP_025993086.1		13	-	-	-	-	-	-	-	-
*CYP307A1* XP_011161205.1		-	3.3	13	-	-	-	-	-	-
*Phtm* (*CYP306A1*) XP_011161459.2		-	4.8	3	2.2	-	-	-	-	-
*CYP315A1* XP_039313250.1		-	6.1	8.9	7.4	-	-	-	-	-
*CYP302A1* XP_011155340.1		-	-	-	-	-	-	-	6.3	17
*Shd* (*CYP314A1*) XP_025995102.1		12	28	13	7.6	10	5.7	3.9	-	20
*CYP18A1* XP_039310694.1		55	12	-	13	-	5.2	19	29	-

The numerals represent overexpression levels, calculated as fold-change ratios by comparing the gene expression levels in different castes and developmental stages to those observed in male alates. “-” indicates no significance compared with male alates.

## Data Availability

The data that support the findings of this study are available from the corresponding author, N.L., upon request.

## Supplementary Materials

The following supporting information can be downloaded at: https://www.mdpi.com/article/10.3390/ijms26073212/s1.

## References

[1] Werck-Reichhart D., Feyereisen R. (2000). Cytochromes P450: A success story. Genome Biol..

[2] Abdelmonem B., Abdelaal N.M., Anwer E.K.E., Rashwan A.A., Hussein M.A., Ahmed Y.F., Khashana R., Hanna M.M., Abdelnaser A. (2024). Decoding the Role of CYP450 Enzymes in Metabolism and Disease: A Comprehensive Review. Biomedicines.

[3] Dermauw W., Leeuwen T., Feyereisen R. (2020). Diversity and evolution of the P450 family in arthropods. Insect Biochem. Mol. Biol..

[4] Sharifiana S., Homaeia A., Kamrani E., Etzerodt T., Pateld S. (2020). New insights on the marine cytochrome P450 enzymes and their biotechnological importance. Int. J. Biol. Macromol..

[5] Chakraborty P., Biswas A., Dey S., Bhattacharjee T., Chakrabarty S. (2023). Cytochrome P450 Gene Families: Role in Plant Secondary Metabolites Production and Plant Defense. J. Xenobiot..

[6] Feyereisen R. (1999). Insect P450 enzymes. Annu. Rev. Entomol..

[7] Feyereisen R., Iatrou K., Gill S. (2005). Insect cytochrome P450. Comprehensive Molecular Insect Science.

[8] Scott G.J., Liu N. (2008). Insect cytochrome P450s: Thinking beyond detoxification. Recent Advances in Insect Physiology, Toxicology and Molecular Biology.

[9] Stipp M.C., Acco A. (2021). Involvement of cytochrome P450 enzymes in inflammation and cancer. Chemother. Pharmacol..

[10] Kweon O., Kim S.J., Kim J.H., Nho S.W., Bae D., Chon J., Hart M., Baek D.-H., Kim Y.-C., Wang W. (2020). CYPminer: An automated cytochrome P450 identification, classification, and data analysis tool for genome data sets across kingdoms. BMC Bioinform..

[11] Wen Z., Scott J.G. (2001). Cytochrome P450 *CYP6L1* is specifically expressed in the reproductive tissues of adult male German cockroaches, *Blattella germanica* (L.). Insect Biochem. Mol. Biol..

[12] Kasai S., Tomita T. (2003). Male specific expression of a cytochrome P450 (*Cyp312a1*) in *Drosophila melanogaster*. Biochem. Biophys. Res. Commun..

[13] Iga M., Kataoka H. (2012). Recent Studies on Insect Hormone Metabolic Pathways Mediated by Cytochrome P450 Enzymes. Biol. Pharm. Bull..

[14] Nouzova M., Edwards M.J., Michalkovaa V., Ramireze C.E., Ruiza M., Areiza M., DeGennaroa M., Fernandez-Limae F., Feyereisen R., Jindrah M. (2021). Epoxidation of juvenile hormone was a key innovation improving insect reproductive fitness. Proc. Natl. Acad. Sci. USA.

[15] Tarver M.R., Zhou X., Scharf M.E. (2010). Socio-environmental and endocrine influences on developmental and caste-regulatory gene expression in the eusocial termite *Reticulitermes flavipes*. BMC Mol. Biol..

[16] Hoffmann K.H. (1997). Ecdysteroids in adult females of a “walking worm”: Euperipatoides leuckartii (Onychophora, Peripatopsidae). Invertebr. Reprod. Dev..

[17] Hoffmann K., Gowin J., Hartfelder K., Korb J. (2014). The Scent of Royalty: A p450 gene signals reproductive status in a social insect. Mole. Biol. Evol..

[18] Psalti M.N., Libbrecht R., Starr C.K. (2021). Caste Differentiation. Encyclopedia of Social Insects.

[19] Orr S.E., Goodisman M.A.D. (2023). Social insect transcriptomics and the molecular basis of caste diversity. Curr. Opin. Insect Sci..

[20] Johnson M.R., Evans J.D., Robinson G.E., Berenbaum M.R. (2009). Changes in transcript abundance relating to colony collapse disorder in honey bee (*Apis mellifera*). Proc. Natl. Acad. Sci. USA.

[21] Richards S., Gibbs R.A., Weinstock G.M., Brown S.J., Denell R., Beeman R.W., Gibbs R., Bucher G., Friedrich M., Grimmelikhuijzen C. (2008). The genome of the model beetle and pest *Tribolium castaneum*. Nature.

[22] Richard G., Jaquiéry J., Le Trionnaire G. (2021). Contribution of epigenetic mechanisms in the regulation of environmentally induced polyphenism in insects. Insects.

[23] Guo B., Mashilingi S.K., Naeem M., Jie C., Zhou Z., Ding G., Huang J., An J. (2024). Differential gene expression responsible for caste determination at both larval and adult stages of Bombus terrestris. Apidologie.

[24] Liu N., Zhang L. (2004). *CYP4AB1*, *CYP4AB2*, and *Gp-9* gene overexpression associated with workers of the red imported fire ant, *Solenopsis invicta Buren*. Gene.

[25] Walsh A.T., Triant D.A., Le Tourneau J.J., Shamimuzzaman M., Elsik C.G. (2022). Hymenoptera genome database: New genomes and annotation datasets for improved GO enrichment and orthologue analyses. Nucleic Acids Res..

[26] Wurm Y., Wang J., Riba-Grognuz O., Corona M., Nygaard S., Hunt B.G., Ingram K.K., Falquet L., Nipitwattanaphon M., Gotzek D. (2011). The genome of the fire ant *Solenopsis invicta*. Proc. Natl. Acad. Sci. USA.

[27] Konorov E.A., Belenikin M.S. (2018). Prediction of the ligands of the CYP9e subfamily of ant cytochrome P450 with the ChEBI ontologies of chemical and biological characteristics. Russ. J. Bioorg. Chem..

[28] Feyereisen R. (2020). Origin and evolution of the CYP4G subfamily in insects, cytochrome P450 enzymes involved in cuticular hydrocarbon synthesis. Mol. Phylogenet. Evol..

[29] Hlavica P. (2011). Insect cytochromes P450: Topology of structural elements predicted to govern catalytic versatility. J. Inorg. Biochem..

[30] Wu Y., Zheng H., Corona M., Pirk C., Meng F., Zheng Y., Hu F. (2017). Comparative transcriptome analysis on the synthesis pathway of honey bee (Apis mellifera) mandibular gland secretions. Sci. Rep..

[31] Malka O., Karunker I., Yeheskel A., Morin S., Hefetz A. (2009). The gene road to royalty—Differential expression of hydroxylating genes in the mandibular glands of the honeybee. FEBS J..

[32] Jing T.X., Wang D.F., Ma Y.P., Zeng L.L., Meng L.W., Zhang Q., Dou W., Wang J.J. (2020). Genome-wide and expression-profiling analyses of the cytochrome P450 genes in Bactrocera dorsalis (Hendel) and screening of candidate P450 genes associated with malathion resistance. Pest Manag. Sci..

[33] Guittard E., Blais C., Maria A., Parvy J.P., Pasricha S., Lumb C., Lafont R., Daborn P.J., Dauphin-Villemant C. (2011). CYP18A1, a key enzyme of Drosophila steroid hormone inactivation, is essential for metamorphosis. Dev. Biol..

[34] Rewitz K.F., Rybczynski R., Warren J.T., Gilbert L.I. (2006). The Halloween genes code for cytochrome P450 enzymes mediating synthesis of the insect molting hormone. Biochem. Soc. Trans..

[35] Zhang Y., Tan Q., Jin L., Li G. (2024). Molecular characterization of the cytochrome P450 enzyme CYP18A1 in *Henosepilachna vigintioctopunctata*. Arch. Insect Biochem. Physiol..

[36] Li Z., Ge X., Ling L., Zeng B., Xu J., Aslam A.F.M., You L., Palli S.R., Huang Y., Tan A. (2023). CYP18A1 regulates tissue-specific steroid hormone inactivation in Bombyx mori. Insect Biochem. Mol. Biol..

[37] Helvig C., Koener J.F., Unnithan G.C., Feyereisen R. (2004). CYP15A1, the cytochrome P450 that catalyzes epoxidation of methyl farnesoate to juvenile hormone III in cockroach corpora allata. Proc. Natl. Acad. Sci. USA.

[38] Huang Y., Wang Z., Zha S., Wang Y., Jiang W., Liao Y., Song Z., Qi Z., Yin Y. (2016). De novo transcriptome and expression profile analysis to reveal genes and pathways potentially involved in cantharidin biosynthesis in the blister beetle Mylabris cichorii. PLoS ONE.

[39] Huang Y., Shen L., Du F., Wang Z., Yin Y. (2024). Functional studies of McSTE24, McCYP305a1, and McJHEH, three essential genes act in cantharidin biosynthesis in the blister beetle (Coleoptera: Meloidae). Insect Sci..

[40] Gilbert L.I. (2004). Halloween genes encode P450 enzymes that mediate steroid hormone biosynthesis in *Drosophila melanogaster*. Mol. Cell Endocrinol..

[41] Petryk A., Warren J.T., Marques G., Jarcho M.P., Gilbert L., Kahler J., Parvy J.-P., Li Y., Dauphin-Villemant C., O’Connor M.B. (2003). Shade is the Drosophila P450 enzyme that mediates the hydroxylation of ecdysone to the steroid insect molting hormone 20-hydroxyecdysone. Proc. Natl. Acad. Sci. USA.

[42] Niwa R., Matsuda T., Yoshiyama T., Namiki T., Mita K., Fujimoto Y., Kataoka H. (2004). CYP306A1, a cytochrome P450 enzyme, is essential for ecdysteroid biosynthesis in the prothoracic glands of Bombyx and Drosophila. J. Biol. Chem..

[43] Kang J., Kim J., Choi K. (2011). Novel cytochrome P450, *cyp6a17*, is required for temperature preference behavior in Drosophila. PLoS ONE.

[44] Zhang X., Yuan D., Ding L., Li P., Li F., Liu X. (2013). Expression of cytochrome P450 *CYP6B6* in the different developmental stages of the insect *Helicoverpa armigera* (Lepidoptera: Noctuidae). Eur. J. Entomol..

[45] Christesen D., Yang Y.T., Somers J., Robin C., Sztal T., Batterham P., Perry T. (2017). Transcriptome analysis of Drosophila melanogaster third instar larval ring glands points to novel functions and uncovers a cytochrome p450 required for development. G3 Genes Genomes Genet..

[46] Fu C., Xiong J., Miao W. (2009). Genome-wide identification and characterization of cytochrome P450 monooxygenase genes in the ciliate Tetrahymena thermophila. BMC Genom..

[47] Terrapon N., Li C., Robertson H.M., Ji L., Meng X., Booth W. (2014). Molecular traces of alternative social organization in a termite genome. Nat. Commun..

[48] Starkey J., Tamborindeguy C. (2023). Molecular mechanisms of task allocation in workers of the red imported fire ant, *Solenopsis invicta*. Insectes Sociaux.

[49] Tarver M.R., Coy M.R., Scharf M.E. (2012). *Cyp15F1*: A novel cytochrome P450 gene linked to juvenile hormone-dependent caste differention in the termite Reticulitermes flavipes. Arch. Insect Biochem. Physiol..

[50] Zheng Y., Zhang W., Xiong Y., Wang J., Jin S., Qiao H., Jiang S., Fu H. (2023). Dual roles of *CYP302A1* in regulating ovarian maturation and molting in *Macrobrachium nipponense*. J. Steroid Biochem. Mol. Biol..

[51] Zhang S., Liu X., Zhu B., Yin X., Du M., Song Q., An S. (2014). Identification of differentially expressed genes in the pheromone glands of mated and virgin Bombyx mori by digital gene expression profiling. PLoS ONE.

[52] Zhang Y.N., Xia Y.H., Zhu J.Y., Li S.Y., Dong S.L. (2014). Putative pathway of sex pheromone biosynthesis and degradation by expression patterns of genes identified from female pheromone gland and adult antenna of Sesamia inferens (Walker). J. Chem. Ecol..

[53] Hartfelder K. (2000). Insect juvenile hormone: From “status quo” to high society. Braz. J. Med. Biol. Res..

[54] Yaguchi H., Masuoka Y., Inoue T., Maekawa K. (2015). Expressions of juvenile hormone biosynthetic genes during presoldier differentiation in the incipient colony of Zootermopsis nevadensis (Isoptera: Archotermopsidae). Appl. Entomol. Zool..

[55] Konopova B., Smykal V., Jindra M. (2011). Common and distinct roles of juvenile hormone signaling genes in metamorphosis of holometabolous and hemimetabolous insects. PLoS ONE.

[56] Rahman M.M., Franch-Marro X., Maestro J.L., Martin D., Casali A. (2017). Local Juvenile Hormone activity regulates gut homeostasis and tumor growth in adult Drosophila. Sci. Rep..

[57] Zhou X., Song C., Grzymala T.L., Oi F.M., Scharf M.E. (2006). Juvenile hormone and colony conditions differentially influence cytochrome P450 gene expression in the termite *Reticulitermes flavipes*. Insect Mol. Biol..

[58] Rewitz K.F., Rybczynski R., Warren J.T., Gilbert L.I. (2006). Development expression of Manduca shade, the P450 mediating the final step in molting hormone synthesis. Mol. Cell. Endocr..

[59] Namiki T., Niwa R., Sakudoh T., Shirai K.I., Takeuchi H., Kataoka H. (2005). Cytochrome P450 *CYP307A1*/Spook: A regulator for ecdysone synthesis in insects. Biochem. Biophys. Res. Commun..

[60] Rewitz K.F., Gilbert L.I. (2008). Daphnia Halloween genes that encode cytochrome P450s mediating the synthesis of the arthropod molting hormone: Evolutionary implications. BMC Evol. Biol..

[61] Petralia R.S., Vinson S.B. (1979). Developmental morphology of larvae and eggs of the imported fire ant, *Solenopsis invicta*. Ann. Entomol. Soc. Am..

[62] Liu N., Scott J.G. (1998). Increased transcription of *CYP6D1* causes cytochrome P450-mediated insecticide resistance in house fly. Insect Biochem. Mol. Biol..

[63] Livak C.T., Ririe K.M., Andrew R.V., David D.A., Gundry R.A., Balis U.J. (1997). The LightCyclerTM: A microvolume multisample fluorimeter with rapid temperature control. BioTechniques.

[64] Livak K.J., Schmittgen T.D. (2001). Analysis of relative gene expression data using real-time quantitative PCR and the 2-ΔΔCT method. Melthods.

[65] Valles S.M., Pereira R.M. (2003). Use of ribosomal DNA sequence data to characterize and detect a neogregarine pathogen of *Solenopsis invicta* (Hymenoptera: Formicidae). J. Invertebr. Pathol..

[66] Strode C., Steen K., Ortelli F., Ranson H. (2006). Differential expression of the detoxification genes in the different life stages of the malaria vector *Anopheles gambiae*. Insect Mol. Biol..

