# Supplementary material for: Genome-Wide Profiling of P450 Gene Expression Reveals Caste-Specific and Developmental Patterns in Solenopsis invicta
Source: Int J Mol Sci. 2025 Mar 30;26(7):3212. doi: 10.3390/ijms26073212 (PMC11989672; doi:10.3390/ijms26073212)
Supplement: Supplementary file 1 [file ijms-26-03212-s001.zip › ijms-3480221-supplementary.pdf]

Table S1 The Real-time qRT-PCR Primers of P450 Genes

NCBI Gene Name	Accession #	NCBI Accession	New Transcript ID	Gene Name*	Forward Primers	Reverse Primers
20-hydroxyecdysone isoform X4	XP_025995102.1	LOC105204717	XM_039458618.1	CYP314A1	AGATTAGCGGAAGCGGTCAGGATT	TGTTTCTCCAACATGGTCGCCTCT
CYP12A2	XP_011164781.2	LOC105199391	XM_011166479.3	CYP12K1	ATACGACCGGTAACGTGTTCAGCA	TCCTTGATGCAGGCCTTAGCGTAT
CYP18A1	XP_039310694.1	LOC105196981	XM_039454761.1	CYP18A1	AACTCAATGGAACCGACGGACTCA	TTTCCATGCCAGCTGAGAAGAGGT
CYP301A1	XP_011175761.2	LOC105207814	XM_011177459.3	CYP301A1	CGTTGCAGAACGACACCGTCATTT	TCTTTGAGCCACCTCGCAGGTTTA
CYP302A1	XP_011155340.1	LOC105192796	XM_011157038.3	CYP302A1	TGTGGGTATGGCGTGTGATATGCT	ACAAAGCCGATGCCTCCGATCTTA
CYP304A1	XP_025988557.2	LOC113003430	XM_026132772.2	CYP369A1	TCGGCTATGCGCAGGAGAAACTTA	TGGCTCACCCTCAACGAAGAAGAA
CYP305A1	XP_025994312.2	LOC105200638	XM_026138527.2	un-named	GGACAACACATGGCCTTTCTGCAT	TTCCAAGGACGCCCATCGTACTTT
CYP305A1	XP_039315195.1	LOC105203155	XM_039459261.1	CYP305D3v1	AACAGAGACCAAAGGACTGTCCAC	TTGCTGAGATGCAGAAAGGCCA
CYP306A1	XP_011161459.2	LOC105196980	XM_039454769.1	CYP306A1	TTTGCCATGGATTGATCCAGCACG	AGAGCCCATTCGTAAGCCACAGAT
CYP307A1	XP_011161205.1	LOC105196791	XM_011162903.3	CYP307B1	ATCATCTCCTCTTCTGCGGCAACA	TCGCAACATCTCTCTTCGCGTCTT
CYP315A1	XP_039313250.1	LOC105207575	XM_039457317.1	CYP315A1	AAACGATGCACGTGCCAGTTTACC	ACCTCCGCCAACGTAAGAGACATT
CYP49A1 isoform X1	XP_011175661.2	LOC105207736	XM_026134053.2	CYP301B1	TGGGAGACAGGATTTGCTACGACA	GCAGAAATCGCCAAGTGTTGCCTA
CYP4AA1	XP_039312209	LOC105198270	XM_039456275.1	CYP4AA6	TCCGAAACAAGGCATCCATTTGCC	ATCCAAAGTCCGCCATGTGCTCTA
CYP4AA1	XP_039308853.1	LOC120358499	XM_039452919.1	CYP4AA7	TCTTTGTTTGCAAGGACACTCGGG	CAGCGCTGAATGGTATACAGGCAT
CYP4AA1	XP_039306960.1	LOC105206700	XM_039451026.1	un-named	GCATCGATGTGCCTACATGCCTTT	TCTTTGCCGAGGACCGGTTCTAAT
CYP4C1	XP_025990768.2	LOC105202834	XM_026134983.2	un-named	ATAGCGCTTGTACATCGGAACCCA	TCCTGTTGAGCGAATCGTATGCCT
CYP4C1	XP_011165193.2	LOC105199689	XM_011166891.2	CYP4BW11	ATCCGGATCGCTTCCTTCCAGAAA	AGGATAGCGCCACCGTACTTGATT
CYP4C1	XP_025990783.2	LOC105199861	XM_026134998.2	un-named	AAGTTGACACGTTTATGGCTGCGG	ACACTAGCCGGTGTCTCCGAATTT
CYP4C1	XP_025990771.2	LOC105202818	XM_026134986.2	CYP4BW9	TGGACACTCTTCCTCTTGGGCAAT	AATTGCGGCAGCTGCTTTACACTG
CYP4C1	XP_011169811.1	LOC105202822	XM_011171509.3	CYP4BW13	AGGCATGAGATTCGCTCAACAGGA	ACCGTATCGGATCGTGTCCAATGT
CYP4C1	XP_025989496.1	LOC105203087	XM_026133711.2	un-named	AGCACCATGGTTAGGAAACGGACT	TATGCTGAAATGGAACGTTGGCCC
CYP4C1 isoform X1	XP_039307300.1	LOC105193889	XM_039451366.1	CYP4AB26	TCGTCATCCCGATGATTTGGAGGT	TTGGAGCCTGCACTGGTAAGAAGA
CYP4C1 isoform X1	XP_039314988.1	LOC105199870	XM_039459055.1	CYP4BW12	CTGAACGGCAATTGCGAAACGACT	ATCGGTCAAGGGAGAACCGTCTTT
CYP4C1 isoform X1	XP_039314235.1	LOC105206429	XM_039458301.1	un-named	TGCTGTGATCGTGTCTGAGAAAGC	ACGTATATCCACACCCATTGCCGT
CYP4C1 isoform X1	XP_039312577.1	LOC105203884	XM_039456650.1	CYP4AB75	TGGTGCCTTCTGGAACAATACTGC	TCGTGGTCCTGCGCTAAATGGTAA
CYP4C1 isoform X2	XP_025993040.2	LOC105194270	XM_026137255.2	CYP4CY4	TCGTGTACTTTGCCAGAGGGATGT	TCTGAGACCCGTGCCAAATGGAAT
CYP4C1 isoform X3	XP_039309284.1	LOC105207342	XM_039453350.1	un-named	TGCAGTAGCTGTATGTTGGGCACT	TCATCCAGCTGCTCTTTAGTGGCT
CYP4C1 isoform X3	XP_011172817	LOC105205216	XM_039450954.1	CYP4AB77	CATTTAGTGCTGGTCCGCGGAAAT	ACGATGTGAATCAAGAGGACGGCA
CYP4C1-like	XP_039307664.1	LOC105200667	XM_039451730.1	un-named	AGATTCAGTGACACCAGCTAGCCA	AAGAGATCAACGGGACGCTTGGTA
CYP4C1-like isoform X2	XP_039306249.1	LOC113003574	XM_039450315.1	un-named	TAATCGATGAGGGCGAGAGCATGA	TACCCATCCGGTAAACGGCTTT
CYP4C21	XP_025991592.2	LOC105202785	XM_026135807.2	CYP4SJ1	AGAAACACCGATGTCTGCACCAGT	CGGCCAAGTATAGGAATAGCAGGA
CYP4C3isoform X1	XP_011157419.1	LOC105194269	XM_039446518.1	CYP4CY5v1	CCTATTGGTCCAAGCCGTTGGAAT	TCTGAGTCCCGTGCCAAATGGAAT

CYP4G15	XP_011156580.1	LOC105193720	XM_011158278.3	CYP4G232	GCTCGCGTGCATGAAGAATTGGAT	TGACCTTTCTGGCAATCCACGGTA
CYP4G15	XP_011170516.1	LOC105203423	XM_039448232.1	CYP4G245	ATGACGACCTTCAAGATGCACCGA	TCAGCTTCAGCATGGCGTATTTGC
CYP4G15 isoform X2	XP_039304511.1	LOC105203564	XM_039448577.1	un-named	TTGCACGAGAAGTGACGGAAGACT	CCGGATCAAACACTAATGGATTCGGC
CYP6A1	XP_011173392.2	LOC105205662	XM_011175091.3	CYP6AS164	TAAGGATGAGGCTGTCACCCGTTT	ATTTATGCCGAAAGCGCAGCTACC
CYP6A13	XP_011172186.2	LOC105204693	XM_011173884.3	CYP336A43	TTTGATGGCCGAATTGGAACGAGC	ACCGATGTAGCTCATTACGCTGCT
CYP6A13	XP_011164185.1	LOC105199000	XM_011165883.3	CYP6BC8	TCGTCGATCCGAGCGTTACCAAAT	AATTCGATTTCCTTCGCCGCTTGG
CYP6A14	XP_011170312.2	LOC105203236	XM_011172010.3	CYP6BE16	ACCGAATAACGAGTACCGAGTCCA	AGTCGGTGACTGATTGTGGTGTGA
CYP6A14	XP_011165642.3	LOC105199991	XM_011167340.3	CYP6BE19	TGATGCAGCTCATGGAGAAAGGCT	GAAGCCAGCTGCGAAGAAGACAAA
CYP6A14	NP_001306576.1	LOC105195645	NM_001319647.1	CYP6AS161	TTCACTTGTTGCTGAACTGTGCCG	ACACGATCCGATCACATCAACGGT
CYP6A14	XP_011159731.1	LOC105195822	XM_011161429.3	CYP6AS163	CGTTGTAGGTGTGTTTTTAAGAGG	ATCTAACAGCGTCGAGCCTGAAGA
CYP6A14	XP_039301776.1	LOC105196480	XM_039445842.1	CYP6BE20	TTGCGTTCTTCATAGCTGGCTTCG	CGTCGATTTCCTCCCGAAGTTTGT
CYP6A14	XP_025997129.2	LOC105198095	XM_026141344.2	CYP6BE22	CGCATCGAAGCGTCACCAAATTCT	CGGGCTTGTTGTCATTGTGGCTAT
CYP6A14	XP_039306285.1	LOC105202192	XM_039450351.1	un-named	AATTTCGACCCTGAGCGCTTCAAC	CAATTTCGTGGACCTTGCCCGAAT
CYP6A14 isoform X1	XP_025992592.1	LOC105203238	XM_039456794.1	CYP6BE15	AGGCATCCTTACGCCTATATGCCA	ACAGAAGACTCACGAGTGCAACCT
CYP6A14 isoform X2	XP_011164433.3	LOC105199166	XM_011166131.3	CYP6BE13	TCGCGTACCAAAGGGAACGCTAAT	AGTAGCCAAATCGCGAACCAATGC
CYP6A17 isoform X1	XP_039310900.1	LOC113003992	XM_039454966.1	un-named	TTTGGCTGGCTACGAAACATCAGC	GCCTCGTACGATATCTCACCATGTTT
CYP6A3	XP_039302616.1	LOC105207889	XM_039446682.1	un-named	AGGAGGTGATGTCCGTGCTAAACA	ATGACCACTGCTGCGGGTATCATT
CYP6J1	XP_011172133.1	LOC105204661	XM_011173831.3	CYP336A45	TCTGTTCGAGACCGTCAAGCAAGT	CATTGTCATCGAAGCACAAGCCGT
CYP6K1	XP_011158950.2	LOC105195279	XM_026130942.2	CYP6ES4	CTTTGGCGTTCGGCATCAGCATAA	CGCGAAAGTAATTCGCGGGTTGTT
CYP6K1	XP_039315612.1	LOC105197535	XM_039459678.1	un-named	TGATCCGCGATCGGGAAATCGTTA	TTGCCCGATGTGAATGTTGGTGTC
CYP6k1	XP_011175846.1	LOC105207882	XM_039446987.1	CYP6AQ58	CGACGCCTTTCTTCGGCAATTTCA	AATACGTAGAAACCCGCGTACGGT
CYP6K1 isoform X1	XP_011162032.2	LOC105197404	XM_039459680.1	CYP6AQ59	AATCGGCATTACTGCCTACGGACT	ATACCGGTCCAACGAACTAGCGTT
CYP6K1-like	XP_039312723.1	LOC105199165	XM_039456789.1	CYP6BE14	TCTGAAGGAGTGTGGCGAAGAACT	GCGGCTGACATGATTACGTCTGTT
CYP6L1-like	XP_039305821.1	LOC120357919	XM_039449887.1	un-named	TATTCCGGTGCTTGGATTGCATCG	TGGCTTCGGTGAAACGTTCCAGAT
CYP9e2	XP_011155385	LOC105192830	XM_011157083.3	CYP9P26	AGGAGAAACCGTTAAAGCGCGAGA	CCAGTTCATGAGCAGCGAAACACA
CYP9E2	XP_011158271.1	LOC105194856	XM_011159969.3	un-named	GCTTCGACGCTGATGTGCTTTGTT	TTTGCGACAGCGAATCGTAGGAGA
Cyp9E2	XP_011160035.1	LOC105196017	XM_011161733.3	CYP9R73	GACGTAATTGCCTCATGCGCCTTT	AAACAGACGTACGAGTGTCGGCAT
CYP9E2-like	XP_039307038.1	LOC120358161	XM_039451104.1	CYP9R70	TTGAGTCCGGCTTTCACATCCAGT	TTAACACCAAAGGCACAAGTGGCG
CYP9E2-like	XP_039306889.1	LOC105205218	XM_039450955.1	CYP9P28	AAGGACTTCGAACACTTCCCGGAT	ACTTCATCTTGCTGGCGGTGAAAG
methyl farnesoate epoxidase	XP_025993632.2	LOC105201474	XM_026137847.2	CYP15A1	TTCTGTTCACCGAAGGCTCCGAAT	CGAGCTTCCATGGCCAATTGTTGT
methyl farnesoate epoxidase	XP_025993086.1	LOC105202927	XM_026137301.2	CYP303A1	TTTGGGCTGTGCGTTGGAAATAGC	AATAACGATGCGATCCTGGCCGAT
methyl farnesoate epoxidase	XP_011156577.1	LOC105193715	XM_011158275.3	CYP343A1	CATCGTCGGCTTGAAACTTGGCAT	TCACCTCCCGTTCGAAGTTTGTGT
Un-named	XP_039307552.1	LOC113003292	XM_039451618.1	CYP9P29	ACCAGGTTGCAAGAGTGTGATCCT	TGCCGATACACTTTCTGGGTCCAT
Un-named	XP_039312260.1	LOC105198269	XM_039456326.1	un-named	ACTCTCGCGTCAGGAAACTCAACA	TAGGCGATTGGCGTACTCACCAAA

Un-named	XP_039301751.1	LOC105197403	XM_039445817.1	CYP6AQ60	ACATGGTGGTGTCCGAAACATTGC	TCGATATATAAACCGGCGTGCCCT
Un-named	XP_025995200.1	LOC105200598	XM_026139415.2	un-named	TACCAACTCGACCGCGATGTCTTT	ACGACACTGCTCCATTCGACTCTT

*From P450 nomenclature Committee, http://drnelson.uthsc.edu/CytochromeP450.html (Dr. David Nelson personal communication).

Table S2. Differential Expression of P450 Genes in Female and Male Alates of *S. invicta*

Gene family	NCBI Gene Name	Accession #	NCBI Accession	New Transcript ID	Gene Name*	Male alate	Female alate	Ratio	SE
CYP18	CYP18A1	XP_039310694.1	LOC105196981	XM_039454761.1	CYP18A1	1.00	28.67±13.34	28.67	13.34
CYP304	CYP304A1	XP_025988557.2	LOC113003430	XM_026132772.2	unnamed	1.00	6.17±2.48	6.17	2.48
CYP305	CYP305A1	XP_025994312.2	LOC105200638	XM_026138527.2	unnamed	1.00	6.52±2.10	6.52	2.10
	CYP305A1	XP_039315195.1	LOC105203155	XM_039459261.1	CYP305D3v1	1.00	5.21±0.98	5.21	0.98
CYP4	CYP4C1 isoform X2	XP_025993040.2	LOC105194270	XM_026137255.2	CYP4CY4	1.00	23.96±5.15	23.96	5.15
	CYP4G15	XP_011170516.1	LOC105203423	XM_039448232.1	CYP4G245	1.00	16.86±0.73	16.86	0.73
	CYP4C1 isoform X1	XP_039314235.1	LOC105206429	XM_039458301.1	unnamed	1.00	14.05±0.68	14.05	0.68
	CYP4G15	XP_011156580.1	LOC105193720	XM_011158278.3	CYP4G232	1.00	5.73±0.19	5.73	0.19
	CYP4C1 isoform X1	XP_039312577.1	LOC105203884	XM_039456650.1	CYP4AB75	1.00	5.42±0.92	5.42	0.92
	CYP4C1-like	XP_039307664.1	LOC105200667	XM_039451730.1	unnamed	1.00	4.67±0.29	4.67	0.29
	CYP4C1	XP_025990783.2	LOC105199861	XM_026134998.2	unnamed	1.00	3.07±1.00	3.07	1.00
	CYP4AA1	XP_039308853.1	LOC120358499	XM_039452919.1	CYP4AA7	1.00	2.76±0.52	2.76	0.52
CYP6	CYP6A14	XP_011165642.3	LOC105199991	XM_011167340.3	CYP6BE19	1.00	8.52±2.42	8.52	2.42
	CYP6A14	XP_011170312.2	LOC105203236	XM_011172010.3	CYP6BE16	1.00	5.20±0.07	5.20	0.07
	CYP6A14 isoform X1	XP_025992592.1	LOC105203238	XM_039456794.1	CYP6BE15	1.00	3.42±1.19	3.42	1.19
	CYP6K1-like	XP_039312723.1	LOC105199165	XM_039456789.1	CYP6BE14	1.00	2.63±0.64	2.63	0.64
CYP302	CYP302A1	XP_011155340.1	LOC105192796	XM_011157038.3	CYP302A1	1.00	6.26±3.32	6.26	3.32
CYP9	CYP9E2-like	XP_039307038.1	LOC120358161	XM_039451104.1	CYP9R70	1.00	0.44±0.09	-2.27	0.09
CYP6	CYP6A3	XP_039302616.1	LOC105207889	XM_039446682.1	unnamed	1.00	0.25±0.10	-4.00	0.10

*From P450 nomenclature Committee, http://drnelson.uthsc.edu/CytochromeP450.html (Dr. David Nelson personal communication).

Table S3. Differential Expression of P450 Genes in Queens Compared to Female Alates of *S. invicta*

Gene family	NCBI Gene Name	Accession #	NCBI Accession	New Transcript ID	Gene Name*	Female alate	Queen	Ratio	SE
CYP305	CYP305A1	XP_025994312.2	LOC105200638	XM_026138527.2	unnamed	6.52±2.10	37.03±11.72	5.68	2.56
CYP4	CYP4AA1	XP_039306960.1	LOC105206700	XM_039451026.1	unnamed	0.87±0.06	5.70±0.08	6.55	0.46
	CYP4C1 isoform X1	XP_039307300.1	LOC105193889	XM_039451366.1	CYP4AB26	1.62±0.03	3.86±0.57	2.38	0.35
	CYP4C1 isoform X1	XP_039314235.1	LOC105206429	XM_039458301.1	unnamed	14.05±0.68	54.94±19.75	3.91	1.42
	CYP4C1 isoform X1	XP_039312577.1	LOC105203884	XM_039456650.1	CYP4AB75	5.42±0.92	16.08±0.26	2.97	0.51
	CYP4C1-like	XP_039307664.1	LOC105200667	XM_039451730.1	unnamed	2.62±0.97	11.09±0.22	4.23	1.57
	CYP4C1 isoform X3	XP_039309284.1	LOC105207342	XM_039453350.1	unnamed	1.97±0.37	23.18±4.33	11.77	3.12
CYP6	CYP6A1	XP_011173392.2	LOC105205662	XM_011175091.3	CYP6AS164	1.48±0.29	62.54±10.31	42.26	10.82
	CYP6A13	XP_039302616.1	LOC105207889	XM_039446682.1	unnamed	0.25±0.10	5.32±1.40	21.28	10.19
	CYP6A13	XP_011164185.1	LOC105199000	XM_011165883.3	CYP6BC8	1.70±0.02	5.59±0.99	3.29	0.58
	CYP6A14 isoform X2	XP_011164433.3	LOC105199166	XM_011166131.3	CYP6BE13	1.72±0.13	13.63±1.44	7.92	1.03
	CYP6A14	NP_001306576.1	LOC105195645	NM_001319647.1	CYP6AS161	0.66±0.20	3.68±0.39	5.58	1.79
	CYP6A14	XP_011159731.1	LOC105195822	XM_011161429.3	CYP6AS163	0.88±0.42	5.71±0.02	6.49	3.10
	CYP6A14	XP_039301776.1	LOC105196480	XM_039445842.1	CYP6BE20	1.29±0.21	5.64±0.12	4.37	0.72
	CYP6A14	XP_025997129.2	LOC105198095	XM_026141344.2	CYP6BE22	0.83±0.03	8.94±3.89	10.77	4.70
	CYP6K1-like	XP_039312723.1	LOC105199165	XM_039456789.1	CYP6BE14	2.63±0.64	10.99±2.21	4.18	1.32
	CYP6L1-like	XP_039305821.1	LOC120357919	XM_039449887.1	unnamed	1.13±0.04	4.37±0.47	3.89	0.44
CYP9	CYP9E2	XP_011158271.1	LOC105194856	XM_011159969.3	unnamed	1.86±0.37	9.33±2.47	5.02	1.66
	CYP9E2-like	XP_039307038.1	LOC120358161	XM_039451104.1	CYP9R70	0.44±0.09	2.48±0.07	5.64	1.16
shd	20-hydroxyecdysone isoform X4	XP_025995102.1	LOC105204717	XM_039458618.1	CYP314A1	1.58±0.23	19.54±0.92	12.37	1.89
CYP302	CYP302A1	XP_011155340.1	LOC105192796	XM_011157038.3	CYP302A1	6.26±3.32	16.76±8.59	2.68	1.97

*From P450 nomenclature Committee, http://drnelson.uthsc.edu/CytochromeP450.html (Dr. David Nelson personal communication).

Table S4 Differential Expression of P450 Genes in Workers Compared to Female Alates of *S. invicta*

Gene family	NCBI Gene Name	Accession #	NCBI Accession	New Transcript ID	Gene Name*	Minim worker	Big worker	Female alate
CYP18	CYP18A1	XP_039310694.1	LOC105196981	XM_039454761.1	CYP18A1	5.16±1.62	18.81±2.31	28.67±13.34
CYP305	CYP305A1	XP_025994312.2	LOC105200638	XM_026138527.2	un-named	20.01±2.30	176.86±20.00	6.52±2.10
CYP4	CYP4C1	XP_025990783.2	LOC105199861	XM_026134998.2	un-named	12.35±7.14	10.17±4.18	3.07±1.00
	CYP4C21	XP_025991592.2	LOC105202785	XM_026135807.2	CYP4SJ1	7.18±3.65	1.09±0.01	0.17±0.14
	CYP4G15 isoform X2	XP_039304511.1	LOC105203564	XM_039448577.1	un-named	8.85±0.96	14.19±1.66	0.32±0.31
CYP6	CYP6A13	XP_011172186.2	LOC105204693	XM_011173884.3	CYP336A43	11.34±4.00	2.91±1.34	1.04±0.77
	CYP6A14	XP_011165642.3	LOC105199991	XM_011167340.3	CYP6BE19	21.93±1.50	10.03±1.00	8.52±2.42
	CYP6A14	NP_001306576.1	LOC105195645	NM_001319647.1	CYP6AS161	2.99±0.83	0.80±0.17	0.66±0.20
	CYP6A14	XP_011159731.1	LOC105195822	XM_011161429.3	CYP6AS163	10.93±2.10	3.35±1.20	0.88±0.42
	CYP6A14	XP_011170312.2	LOC105203236	XM_011172010.3	un-named	16.35±3.80	4.37±0.31	1.83±0.52
	CYP6A14	XP_039301776.1	LOC105196480	XM_039445842.1	CYP6BE20	9.23±1.53	3.19±1.55	1.29±0.21
	CYP6K1 isoform X1	XP_011162032.2	LOC105197404	XM_039459680.1	CYP6AQ59	112.00±30.00	31.12±15.10	0.73±0.36
	CYP6L1-like	XP_039305821.1	LOC120357919	XM_039449887.1	un-named	4.86±2.71	6.44±2.05	1.13±0.04
CYP9	CYP9E2	XP_011160035.1	LOC105196017	XM_011161733.3	CYP9R73	3.87±0.54	1.52±0.56	0.44±0.24
epoxidase	methyl farnesoate epoxidase	XP_025993632.2	LOC105201474	XM_026137847.2	CYP15A1	9.30±2.14	3.16±1.36	0.67±0.12
shd	ecdysone 20-monooxygenase isoform X4	XP_025995102.1	LOC105204717	XM_039458618.1	CYP314A1	5.71±1.53	3.94±2.16	1.58±0.23
un-named	Un-named	XP_039301751.1	LOC105197403	XM_039445817.1	CYP6AQ60	37.09±19.35	2.67±1.10	1.43±0.53

*From P450 nomenclature Committee, http://drnelson.uthsc.edu/CytochromeP450.html (Dr. David Nelson personal communication).

Table S5 Comparison of P450 Gene Expression Between 1st–2nd and 3rd–4th Instar Larvae of *S. invicta*

A. Upregulated P450 Genes in 3rd–4th Compared to 1st–2nd Instar Larvae

Gene family	NCBI Gene Name	Accession #	NCBI Accession	New Transcript ID	Gene Name*	1st-2nd larva	3rd-4th larva	Ratio	SE
CYP305	CYP305A1	XP_039315195.1	LOC105203155	XM_039459261.1	CYP305D3v1	0.56±0.27	254.04±71.4	453.00	100.00
CYP4	CYP4G15	XP_011170516.1	LOC105203423	XM_039448232.1	CYP4G245	0.82±0.30	11.62±1.31	14.17	5.42
	CYP4G15 isoform X2	XP_039304511.1	LOC105203564	XM_039448577.1	unnamed	1.02±0.42	78.93±12.30	77.38	34.07
CYP6	CYP6A1	XP_011173392.2	LOC105205662	XM_011175091.3	CYP6AS164	4.45±1.20	29.75±5.32	6.69	2.16
	CYP6A14	XP_011170312.2	LOC105203236	XM_011172010.3	unnamed	3.79±1.10	14.56±2.24	3.84	1.26
shd	ecdysone 20-monooxygenase isoform X4	XP_025995102.1	LOC105204717	XM_039458618.1	CYP314A1	12.33±2.31	27.63±10.84	2.24	0.97
CYP315A1	CYP315A1	XP_039313250.1	LOC105207575	XM_039457317.1	CYP315A1	1.06±0.27	6.11±1.23	5.76	1.87
phtm/306A1	phantom	XP_011161459.2	LOC105196980	XM_039454769.1	CYP306A1	1.87±0.22	4.77±2.40	2.55	1.32
CYP307	CYP307A1	XP_011161205.1	LOC105196791	XM_011162903.3	CYP307B1	0.58±0.20	3.26±1.13	5.62	2.22

B. Upregulated P450 Genes in 1st–2nd Compared to 3rd–4th Instar Larvae

Gene family	NCBI Gene Name	Accession #	NCBI Accession	New Transcript ID	Gene Name*	3rd-4th larva	1st-2nd larva	Ratio	SE
CYP18	CYP18A1	XP_039310694.1	LOC105196981	XM_039454761.1	CYP18A1	12.4±2.50	55.34±0.61	4.55	0.90
CYP4	CYP4C1-like	XP_039307664.1	LOC105200667	XM_039451730.1	unnamed	18.04±10.37	241.36±46	14.29	2.10
CYP6	CYP6A14	XP_025997129.2	LOC105198095	XM_026141344.2	CYP6BE22	2.25±0.51	65.93±4.60	25.00	6.95
	CYP6A14	XP_011170312.2	LOC105203236	XM_011172010.3	CYP6BE16	4.10±0.36	26.85±4.29	6.67	1.19
	CYP6K1-like	XP_039312723.1	LOC105199165	XM_039456789.1	CYP6BE14	3.80±0.65	24.14±7.43	6.25	2.24
	CYP6A14	NP_001306576.1	LOC105195645	NM_001319647.1	CYP6AS161	0.26±0.01	1.46±0.05	5.56	0.29
	CYP6K1	XP_011175846.1	LOC105207882	XM_039446987.1	CYP6AQ58	0.52±0.04	2.76±0.59	5.26	1.21
	CYP6A13	XP_011172186.2	LOC105204693	XM_011173884.3	CYP336A43	1.05±0.11	5.59±0.26	5.26	0.61
	CYP6A14 isoform X2	XP_011164433.3	LOC105199166	XM_011166131.3	CYP6BE13	9.42±3.58	43.56±0.07	4.55	1.76
	CYP6L1-like	XP_039305821.1	LOC120357919	XM_039449887.1	unnamed	14.19±2.96	44.26±1.85	3.13	0.66
	CYP6A17 isoform X1	XP_039310900.1	LOC113003992	XM_039454966.1	unnamed	14.02±1.85	34.90±0.34	2.50	0.33
epoxidase	methyl farnesoate epoxidase	XP_025993086.1	LOC105202927	XM_026137301.2	CYP303A1	1.83±0.39	12.97±2.42	7.14	2.01

*From P450 nomenclature Committee, http://drnelson.uthsc.edu/CytochromeP450.html (Dr. David Nelson personal communication).

Table S6. Comparison of P450 Gene Expression Between Worker Pupae, Minim Workers, and Big Workers of *S. invicta*

Gene family	NCBI Gene Name	Accession #	NCBI Accession	New Transcript ID	Gene Name*	Minim worker	Big Worker	Worker Pupa
phtm/306A1	phantom	XP_011161459.2	LOC105196980	XM_039454769.1	CYP306A1	1.00±0.05	0.31±0.07	2.98±0.35
CYP307	CYP307A1	XP_011161205.1	LOC105196791	XM_011162903.3	CYP307B1	1.12±0.64	0.45±0.04	12.75±4.85
CYP4	CYP4G15 isoform X2	XP_039304511.1	LOC105203564	XM_039448577.1	unnamed	8.85±0.96	14.19±1.66	362.77±6.40
CYP6	CYP6A14	XP_039301776.1	LOC105196480	XM_039445842.1	CYP6BE20	9.23±1.53	1.00±0.10	28.52±6.21
	CYP6A14	XP_025997129.2	LOC105198095	XM_026141344.2	unnamed	1.83±0.41	0.32±0.03	7.03±0.99
	CYP6A17 isoform X1	XP_039310900.1	LOC113003992	XM_039454966.1	unnamed	2.98±0.53	3.48±0.04	14.78±4.10
	CYP6L1-like	XP_039305821.1	LOC120357919	XM_039449887.1	unnamed	4.86±2.71	6.44±2.05	12.92±1.12
shd	ecdysone 20-monooxygenase isoform X4	XP_025995102.1	LOC105204717	XM_039458618.1	CYP314A1	5.71±1.53	3.94±2.16	12.56±4.83
CYP315A1	CYP315A1	XP_039313250.1	LOC105207575	XM_039457317.1	CYP315A1	1.72±0.16	0.81±0.15	8.91±1.07
CYP49	CYP49A1 isoform X1	XP_011175661.2	LOC105207736	XM_026134053.2	CYP301B1	0.37±0.15	0.29±0.13	2.01±0.33
unnamed	unnamed	XP_025988557.2	LOC113003430	XM_026132772.2	unnamed	7.55±4.89	3.52±1.25	20.42±2.31

Table S7. Comparison of P450 Gene Expression in Worker, Male, and Female Pupae Relative to 3rd–4th Instar Larvae of *S. invicta*

Gene family	NCBI Gene Name	Accession #	NCBI Accession	New Transcript ID	Gene Name*	3rd-4th instar	Worker Pupa	Male alate pupa	Female alate pupa	Ratio	SE
CYP4	CYP4G15 isoform X2	XP_039304511.1	LOC105203564	XM_039448577.1	unnamed	78.93±5.6	358.77±12.40			4.54	0.36
CYP6	CYP6K1-like	XP_039312723.1	LOC105199165	XM_039456789.1	CYP6BE14	3.8±0.65	13.41±2.13			3.53	0.82
	CYP6K1	XP_039315612.1	LOC105197535	XM_039459678.1	unnamed	2.6±0.24	7.81±1.69			3.00	0.71
CYP307	CYP307A1	XP_011161205.1	LOC105196791	XM_011162903.3	CYP307B1	3.26±1.13	12.75±4.85			3.91	1.01
CYP4	CYP4C1	XP_025990771.2	LOC105202818	XM_026134986.2	CYP4BW9	0.06±0.002		0.19±0.06		3.17	1.01
epoxidase	methyl farnesoate epoxidase	XP_011156577.1	LOC105193715	XM_011158275.3	CYP343A1	1.83±0.39		5.34±1.22		2.92	0.52
CYP4	CYP4C1	XP_025990783.2	LOC105199861	XM_026134998.2	unnamed	10.37±3.13			24.21±5.78	2.33	0.78
CYP4	CYP4C1	XP_039309284.1	LOC105207342	XM_039453350.1	unnamed	1.04±0.28			2.71±1.03	2.61	0.75

*From P450 nomenclature Committee, http://drnelson.uthsc.edu/CytochromeP450.html (Dr. David Nelson personal communication).

Table S8. Comparison of P450 Gene Expression Between Male Pupae and Male Alates, and Between Female Pupae and Female Alates of *S. invicta*

A. Comparison of P450 Gene Expression Between Male Pupae and Male Alates

Gene family	NCBI Gene Name	Accession #	NCBI Accession	New Transcript ID	Gene Name*	Male alate	Male pupa	Ratio	SE
phtm/306A1	CYP306A1	XP_011161459.2	LOC105196980	XM_039454769.1	CYP306A1	1.00	2.16±0.01	2.16	0.01
CYP315	CYP315A1	XP_039313250.1	LOC105207575	XM_039457317.1	CYP315A1	1.00	7.36±1.77	7.36	1.77
CYP18	CYP18A1	XP_039310694.1	LOC105196981	XM_039454761.1	CYP18A1	1.00	13.19±2.31	13.19	2.31
CYP305	CYP305A1	XP_025994312.2	LOC105200638	XM_026138527.2	unnamed	1.00	3.53±0.86	3.53	0.86
	CYP305A1	XP_039315195.1	LOC105203155	XM_039459261.1	CYP305D3v1	1.00	52.21±18.35	52.21	18.35
CYP49	CYP49A1 isoform X1	XP_011175661.2	LOC105207736	XM_026134053.2	CYP301B1	1.00	3.82±0.54	3.82	0.54
CYP4	CYP4C1	XP_025990783.2	LOC105199861	XM_026134998.2	unnamed	1.00	16.82±3.74	16.82	3.74
	CYP4C1isoform X3	XP_039309284.1	LOC105207342	XM_039453350.1	unnamed	1.00	18.40±3.21	18.40	3.21
	CYP4C1-like	XP_039307664.1	LOC105200667	XM_039451730.1	unnamed	1.00	30.50±6.08	30.50	6.08
	CYP4C3isoform X1	XP_011157419.1	LOC105194269	XM_039446518.1	CYP4CY5v1	1.00	5.60±1.24	5.60	1.24
	CYP4G15	XP_011170516.1	LOC105203423	XM_039448232.1	CYP4G245	1.00	7.21±2.13	7.21	2.13
	CYP4G15 isoform X2	XP_039304511.1	LOC105203564	XM_039448577.1	unnamed	1.00	8.15±1.87	8.15	1.87
CYP6	CYP6A14	XP_011165642.3	LOC105199991	XM_011167340.3	CYP6BE19	1.00	5.00±1.42	5.00	1.42
	CYP6A14	XP_039301776.1	LOC105196480	XM_039445842.1	CYP6BE20	1.00	4.44±0.31	4.44	0.31
	CYP6A14	XP_025997129.2	LOC105198095	XM_026141344.2	CYP6BE22	1.00	13.82±5.66	13.82	5.66
	CYP6A17isoform X1	XP_039310900.1	LOC113003992	XM_039454966.1	unnamed	1.00	5.84±2.15	5.84	2.15
	CYP6L1-like	XP_039305821.1	LOC120357919	XM_039449887.1	unnamed	1.00	14.23±5.57	14.23	5.57
epoxidase	methyl farnesoate epoxidase	XP_011156577.1	LOC105193715	XM_011158275.3	CYP343A1	1.00	5.34±1.22	5.34	1.22
shd	shade	XP_025995102.1	LOC105204717	XM_039458618.1	CYP314A1	1.00	7.63±4.06	7.63	4.06

B. Comparison of P450 Gene Expression Between Female Pupae and Female Alates

Gene family	NCBI Gene Name	Accession #	NCBI Accession	New Transcript ID	Gene Name*	Female alate	Female alate pupa	Ratio	SE
CYP4	CYP4AA1	XP_039306960.1	LOC105206700	XM_039451026.1	unnamed	0.87±0.06	4.46±0.12	5.13	0.38
	CYP4G15 isoform X2	XP_039304511.1	LOC105203564	XM_039448577.1	unnamed	0.32±0.31	11.07±0.40	34.59	5.54
CYP6	CYP6K1isoform X1	XP_011162032.2	LOC105197404	XM_039459680.1	CYP6AQ59	0.73±0.36	2.27±0.05	3.11	0.54
	CYP6L1-like	XP_039305821.1	LOC120357919	XM_039449887.1	unnamed	1.13±0.04	3.26±1.48	2.88	0.31
shd	shade	XP_025995102.1	LOC105204717	XM_039458618.1	CYP314A1	1.58±0.52	10.43±5.38	6.60	2.04
CYP4	CYP4C1isoform X3	XP_039309284.1	LOC105207342	XM_039453350.1	unnamed	10.89±0.40	5.53±0.01	-2.00	0.01

*From P450 nomenclature Committee, http://drnelson.uthsc.edu/CytochromeP450.html (Dr. David Nelson personal communication).
